# Mesenchymal stem cells used as carrier cells of oncolytic adenovirus results in enhanced oncolytic virotherapy

**DOI:** 10.1038/s41598-019-57240-x

**Published:** 2020-01-16

**Authors:** Khaphetsi Joseph Mahasa, Lisette de Pillis, Rachid Ouifki, Amina Eladdadi, Philip Maini, A-Rum Yoon, Chae-Ok Yun

**Affiliations:** 1 DST/NRF Centre of Excellence in Epidemiological Modelling and Analysis (SACEMA), University of Stellenbosch, Stellenbosch, South Africa; 2 Department of Mathematics and Applied Mathematics, University of Pretoria, Pretoria, South Africa; 3 Department of Mathematics, Harvey Mudd College, Claremont, CA USA; 4 Department of Mathematics, The College of Saint Rose, Albany, NY USA; 5 Wolfson Centre for Mathematical Biology, Mathematical Institute, University of Oxford, Oxford, United Kingdom; 6 Department of Bioengineering, College of Engineering, Hanyang University, Seoul, Republic of Korea

**Keywords:** Computational models, Stem-cell research

## Abstract

Mesenchymal stem cells (MSCs) loaded with oncolytic viruses are presently being investigated as a new modality of advanced/metastatic tumors treatment and enhancement of virotherapy. MSCs can, however, either promote or suppress tumor growth. To address the critical question of how MSCs loaded with oncolytic viruses affect virotherapy outcomes and tumor growth patterns in a tumor microenvironment, we developed and analyzed an integrated mathematical-experimental model. We used the model to describe both the growth dynamics in our experiments of firefly luciferase-expressing Hep3B tumor xenografts and the effects of the immune response during the MSCs-based virotherapy. We further employed it to explore the conceptual clinical feasibility, particularly, in evaluating the relative significance of potential immune promotive/suppressive mechanisms induced by MSCs loaded with oncolytic viruses. We were able to delineate conditions which may significantly contribute to the success or failure of MSC-based virotherapy as well as generate new hypotheses. In fact, one of the most impactful outcomes shown by this investigation, not inferred from the experiments alone, was the initially counter-intuitive fact that using tumor-promoting MSCs as carriers is not only helpful but necessary in achieving tumor control. Considering the fact that it is still currently a controversial debate whether MSCs exert a pro- or anti-tumor action, mathematical models such as this one help to quantitatively predict the consequences of using MSCs for delivering virotherapeutic agents *in vivo*. Taken together, our results show that MSC-mediated systemic delivery of oncolytic viruses is a promising strategy for achieving synergistic anti-tumor efficacy with improved safety profiles.

## Introduction

For most advanced or metastatic tumors, only a limited number of therapeutic options are available for cancer patients. Oncolytic viruses (i.e. viruses that selectively replicate and destroy cancer cells while having limited or no toxicity to normal cells) have emerged as promising novel therapeutic strategy against most advanced types of cancers. Their delivery to tumor sites, however, remains a major obstacle. When oncolytic viruses (OVs) cannot be injected directly into a target tumor, only a limited fraction (usually administered intravenously) manage to migrate and reach the target tumor site. This is often due to antiviral immunity in the blood which rapidly clears the viruses^1,2^. Clinical evidence indicates that even for high doses of intravenous OVs, the efficient systemic delivery of OVs is still limited^3,4^.

To overcome these challenges, several strategies have been explored including the use of cells that have the potential to home in towards the tumor microenvironment as delivery vehicles for OVs^5,6^. Some carrier cells are used as Trojan horses which can internalize the OVs and allow virus replication, but have no role after successful OV delivery in tumor sites^7^.

In recent years, MSCs have been identified as promising vectors for the delivery of anti-cancer agents due to their strong inherent tropism into the tumor microenvironment where they not only constitute cellular components, but also regulate tumor growth^8^. While within the tumor microenvironment, MSCs can interact with tumor cells in several ways which may result in the promotion of tumor growth^9,10^. This mechanisms include suppression of local immune response^11,12^, stimulation of the epithelial–mesenchymal transition^13^, promotion of angiogenesis^10,14^, inhibition of tumor cell apoptosis, and promotion of tumor metastasis^9,15^.

Another important attribute of MSCs which often make them attractive candidates for OVs delivery, is that they support viral replication while loaded with the virus^11,12,16^. MSCs are also known to protect (through internalization) the pre-loaded virus from immune-mediated neutralization during their migration to tumor sites^12,17,18^. Building on this, recent experimental studies show that MSCs not only hide the pre-loaded OVs from immune cells during their trafficking to the tumor site, but they can also suppress the immune response^11,12^. It is, however, not fully understood how MSCs precisely suppress the immune system^11^. In contrast to their tumor promoting abilities, several studies report that MSCs can also suppress tumor growth^9,19^. MSCs can inhibit growth of tumor cells through inhibition of angiogenesis^20^, induction of cell cycle arrest and apoptosis^21^, enhancement of inflammatory infiltration^22^, and inhibition of proliferation-related signaling pathways, such as Wnt^9,23^. Despite that, it is still not fully understood how MSCs facilitate the inhibition of tumor cell growth because no study has precisely indicated which ligand is responsible for the induction of tumor growth suppression^9^. Currently, it is still controversial whether MSCs suppress or promote tumor development^8^. Given the lack of information on the local interactions involved within the tumor microenvironment upon the arrival of OV carrier cells, we investigate whether using MSCs as OV carriers can significantly contribute to tumor cell death (lysis) induced by OVs upon their arrival at the tumor microenvironment.

In spite of these tumor-promotive/suppressive mechanisms, multiple challenges remain to be fully addressed before MSC-based virotherapeutic approaches can be routinely applied in clinical settings. MSCs derived from different tissues in a patient can produce widely varying outcomes in relation to secretion of cytokines and chemokines, and immunomodulatory potential^24–26^. Hence, it becomes difficult to predict how different patients will respond to the MSC-based cell carrier therapies^25^.

Currently, there is a small number of experimental-mathematical models that address the challenge of low delivery of therapeutic agents to the tumor microenvironments. Such models include use of nanoparticles^27–29^ and macrophages^30,31^ to deliver therapeutic drugs to tumor sites. There is, however, no mathematical model that has investigated the use of highly unpredictable mesenchymal stem cells in the presence of active immune response in oncolytic virotherapy. Thus, our modeling approach aims to bridge this gap. In an effort to better understand the current limitations of oncolytic virotherapy and how we might redesign better and successful virotherapies, it is invaluable to adopt an integrated mathematical-experimental approach. Mathematical modeling provides a theoretical framework that can be used both descriptively and predictively to explain the complexity of the tumor-immune-therapy interactions. A quantitative understanding of these interactions would help to design better and successful MSC-based virotherapies. In this contribution, we develop a mathematical model calibrated with our *in vivo* and *in vitro* experiments^32^ to investigate the tumor response to the use of MSCs as cellular delivery vehicles for OVs.

## Materials and Methods

### Experiments: Oncolytic adenovirus delivery by mesenchymal stem cells

The study protocol was in accordance with the declaration of Helsinki. After receiving the informed consent, bone marrow was obtained from healthy donors. All the manufacturing and product testing procedures for hMSC generation were performed using good manufacturing practices (Pharmicell Co. Ltd., Seongnam, Korea). This research protocol was reviewed and approved by Institutional Review Board of Asan Medical Center, Seoul, Korea (2015–1123). All aspects of animal care and treatment were performed in a facility approved by the Association for Assessment and Accreditation of Laboratory Animal Care. All animal studies were performed according to the institutionally approved protocols of University of Utah and Hanyang University. All mice were housed for 1 week for acclimatization, and ad libitum access to food and water was provided. The experiment for assessing MSCs as cell carriers of oncolytic Ads was carried out for both *in vitro* and *in vivo* settings as follows^32^.

### *In vivo* tumor growth analysis

The experimental design of using mesenchymal stem cells (MSCs) as cell carriers of oncolytic Ad and tumor growth data in response to oncolytic Ad has been reported in^32^. The study evaluates the therapeutic efficacy of oAd-loaded MSCs on luciferase-expressing orthotopic Hep 3B tumors which were treated with phosphate buffered saline (PBS), MSCs, oncolytic adenovirus (oAd), and MSCs infected with oAd (oAd-MSCs). The orthotopic hepatocellular carcinoma cancer model was established by injecting 1 × 10^6^ firefly luciferase-expressing Hep 3B cells into the left lobe of the liver in athymic nude mice. At 7 days post-implantation, blood was harvested by retro-orbital bleeding, and the level of AFP was analyzed by enzyme-linked immunosorbent assay (ELISA) according to manufacturer’s instruction. The mice were randomly divided into three groups by serum AFP level and treated with an intravenous injection of PBS, 1 × 10^6^ MSCs, 5 × 10^8^ virus particles (VP) of oncolytic Ad, and oAd-MSC (1 × 10^6^ MSCs infected with 5 × 10^8^ VP of oncolytic Ad) on day 9 and 13 post-tumor cell implantation (*n* = 6 per group). Optical imaging, with an IVIS SPECTRUM instrument, was conducted every week and luciferase activity was quantitatively analyzed with IGOR-PRO Living Image software. A group of tumor-bearing mice that were treated with PBS served as controls.

### Mathematical model

#### Biological assumptions

Since cell migration, or trafficking, occurs across complex multiple cellular networks, we assume, for simplicity, that MSCs have successfully homed in to the tumor sites where they can deliver their therapeutic payloads. Note that in this study, we do not consider the mechanisms that induce MSC migration to tumor sites, but we model the local interactions between MSCs loaded with oncolytic Ads (oAd-MSCs), free oncolytic Ads within the tumor microenvironment, immune cells, and tumor cells. We also assume that the oncolytic Ads are successfully pre-loaded MSCs. Here, MSCs are not only used as Trojan horses, but as cells that can also interact with tumor cells and, possibly, promote tumor growth or induce tumor suppression. Furthermore, we assume that there are other local immune lymphocytes within the tumor microenvironment: Natural killer (NK) cells and activated cytotoxic T lymphocytes (CTLs). While there are more than 10 types of immune cells, we included the main components of the immune system that are relevant to oncolytic virotherapy. The viral-tumor specific T cell (CTL) response contributes to the efficacy of oncolytic Ad therapy by improving tumor oncolysis and mediating a long-term anti-tumor immune response^33^, while NK cells, as part of the innate immune response, are recruited and activated to clear both OVs and infected tumor cells following initial OV treatment^34^. Upon their release from oAd-MSCs, for simplicity, we assume that OVs can only interact with tumor cells at the tumor site, even though it is possible that viruses can also interact with immune cells^35,36^.

Using this new integrated mathematical-experimental framework, our major goals are two-fold: (1) to investigate the efficacy of oAd-MSCs treatment and the immune response to tumor cells. (2) to compare the oAd-MSC dosing regimen (i.e., when MSCs are used as delivery vehicles of oncolytic Ads) with the direct dose of naked oncolytic Ad regime, from a quantitative perspective.

Our model builds upon the following biological assumptions: (a) in the absence of immune response and OVs, tumor growth is characterized by logistic growth dynamics; (b) as part of the innate immunity, NK cells are always present in the tumor microenvironment, even in the absence of tumor cells, while CTLs are present only when a tumor is present; (c) after lysis of MSCs, OVs infect tumor cells. Since not all viruses can successfully infect tumor cells, we assume that free viruses are cleared by the antiviral immune cells within the tumor microenvironment; (d) the oAd-MSCs can only promote or suppress growth of the proliferating uninfected tumor cells since the life-span of infected cells is short (i.e., the virus rapidly lyses the infected cell as modeled in^37^); (d) we assume that the tumor microenvironment and cell populations are spatially homogeneous. (e) We also assume that there is no genetic variability within one cell population. It is known that tumor heterogeneity is important, but in this model, for simplicity, we assume that the model describes the average behaviour of a cell.

#### State variables and parameters

**Figure 1:**
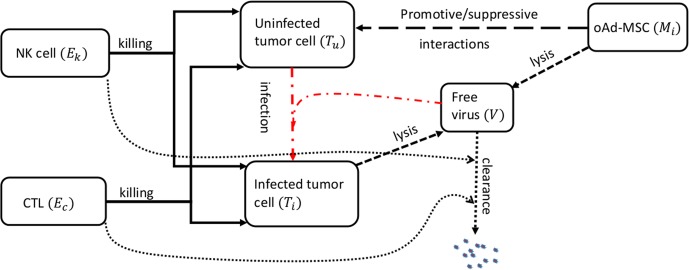
Model interaction network within the tumor microenvironment. Oncolytic Ad carrier cells: MSC loaded with OVs (*M*_*i*_) undergo lysis (Short dash line) resulting in release of free oncolytic Ads (*V*). oAd-MSCs promote/suppress tumor cell proliferation (Long dash line). Uninfected tumor cells (*T*_*u*_) become infected cells (*T*_*i*_) upon successful entry of the virus (Dash dot line). Infected cells also undergo lysis (Short dash line) resulting in more free viruses. Cytotoxic immune cells: Natural killer cells (*E*_*K*_) and cytotoxic T lymphocytes (*E*_*C*_) kill tumor cells (Solid line) and clear free viruses (Round dot line).

The mathematical model is based on the interaction network illustrated in Fig. 1. The independent variable is time, *t*, and the state variables considered in this model are as follows: *T*_*u*_(*t*), the total number of uninfected tumor cells; *T*_*i*_(*t*), the total number of infected tumor cells; *M*_*i*_(*t*), the total number of MSC carriers in the tumor microenvironment (oAd-MSC); *V*(*t*), the total number of virions released within the tumor microenvironment; *E*_*K*_(*t*), the total number of NK cells within the tumor microenvironment; and *E*_*C*_(*t*), the total number of activated CTLs within the tumor microenvironment. The model parameters, together with their units and sources, are summarized in Table S1.

***Key Equations***:

1 $$\begin{array}{ccc}\frac{d{T}_{u}}{dt} & = & {a}_{T}\mathop{\underbrace{(1+\frac{{\delta }_{p/s}\eta {M}_{i}}{{h}_{TM}+{M}_{i}}){T}_{u}(1-\frac{{T}_{u}+{T}_{i}}{{K}_{T}})}}\limits_{{\rm{t}}{\rm{u}}{\rm{m}}{\rm{o}}{\rm{r}}\,{\rm{p}}{\rm{r}}{\rm{o}}{\rm{l}}{\rm{i}}{\rm{f}}{\rm{e}}{\rm{r}}{\rm{a}}{\rm{t}}{\rm{i}}{\rm{o}}{\rm{n}}}-\mathop{\underbrace{{\beta }_{T}({t}_{i}){T}_{u}V}}\limits_{{\rm{i}}{\rm{n}}{\rm{f}}{\rm{e}}{\rm{c}}{\rm{t}}{\rm{i}}{\rm{o}}{\rm{n}}}-\mathop{\underbrace{{\lambda }_{T}{E}_{K}{T}_{u}}}\limits_{{\rm{k}}{\rm{i}}{\rm{l}}{\rm{l}}{\rm{i}}{\rm{n}}{\rm{g}}\,{\rm{b}}{\rm{y}}\,{\rm{N}}{\rm{K}}}\\  &  & -\,D\mathop{\underbrace{[\frac{{E}_{C}}{{T}_{u}+{T}_{i}}]{T}_{u}}}\limits_{{\rm{k}}{\rm{i}}{\rm{l}}{\rm{l}}{\rm{i}}{\rm{n}}{\rm{g}}\,{\rm{b}}{\rm{y}}\,{\rm{C}}{\rm{T}}{\rm{L}}}\end{array}$$

2 $$\frac{d{T}_{i}}{dt}=\mathop{\underbrace{{\beta }_{T}({t}_{i}){T}_{u}V}}\limits_{{\rm{i}}{\rm{n}}{\rm{f}}{\rm{e}}{\rm{c}}{\rm{t}}{\rm{i}}{\rm{o}}{\rm{n}}}-\mathop{\underbrace{{l}_{v}(MOI){T}_{i}}}\limits_{{\rm{d}}{\rm{e}}{\rm{a}}{\rm{t}}{\rm{h}}\,{\rm{b}}{\rm{y}}\,{\rm{l}}{\rm{y}}{\rm{s}}{\rm{i}}{\rm{s}}}-\mathop{\underbrace{{\lambda }_{T}{E}_{K}{T}_{i}}}\limits_{{\rm{k}}{\rm{i}}{\rm{l}}{\rm{l}}{\rm{i}}{\rm{n}}{\rm{g}}\,{\rm{b}}{\rm{y}}\,{\rm{N}}{\rm{K}}}-\mathop{\underbrace{D[\frac{{E}_{C}}{{T}_{u}+{T}_{i}}]{T}_{i}}}\limits_{{\rm{k}}{\rm{i}}{\rm{l}}{\rm{l}}{\rm{i}}{\rm{n}}{\rm{g}}\,{\rm{b}}{\rm{y}}\,{\rm{C}}{\rm{T}}{\rm{L}}}$$

3 $$\frac{d{M}_{i}}{dt}=\mathop{\underbrace{{\xi }_{M}{u}_{M}(t)}}\limits_{{\rm{source}}}-\mathop{\underbrace{{l}_{v}(MOI){M}_{i}}}\limits_{{\rm{death}}\,{\rm{by}}\,{\rm{lysis}}}$$

4 $$\frac{dV}{dt}=\mathop{\underbrace{{\xi }_{V}{u}_{V}(t)}}\limits_{{\rm{source}}}+\mathop{\underbrace{{l}_{v}(MOI){b}_{M}{M}_{i}}}\limits_{{\rm{lysis}}}+\mathop{\underbrace{{l}_{v}(MOI){b}_{T}{T}_{i}}}\limits_{{\rm{lysis}}}-\mathop{\underbrace{\omega V}}\limits_{{\rm{clearance}}}$$

5 $$\frac{d{E}_{K}}{dt}=\mathop{\underbrace{{S}_{{E}_{K}}(t)}}\limits_{{\rm{source}}}-\mathop{\underbrace{{r}_{K}{\lambda }_{T}{E}_{K}({T}_{u}+{T}_{i})}}\limits_{{\rm{inactivation}}}-\mathop{\underbrace{{\mu }_{K}{E}_{K}}}\limits_{{\rm{natural}}\,{\rm{death}}}$$

6 $$\frac{d{E}_{C}}{dt}=\mathop{\underbrace{\gamma {E}_{C}\frac{({T}_{u}+{T}_{i})}{{h}_{T}+{T}_{u}+{T}_{i}}}}\limits_{{\rm{recruitment}}}-\mathop{\underbrace{{r}_{C}{E}_{C}({T}_{u}+{T}_{i})}}\limits_{{\rm{inactivation}}}-\mathop{\underbrace{{\mu }_{C}{E}_{C}}}\limits_{{\rm{natural}}\,{\rm{death}}}$$

where

7 $$D[x]=\alpha \frac{{x}^{l}}{{h}_{{E}_{C}}+{x}^{l}}.$$

Note that

8 $$\{\begin{array}{c}\begin{array}{cc}\eta  < 0 & {\rm{i}}{\rm{f}}\,{\rm{o}}{\rm{A}}{\rm{d}}{\textstyle \mbox{--}}{\rm{M}}{\rm{S}}{\rm{C}}\,{\rm{s}}{\rm{u}}{\rm{p}}{\rm{p}}{\rm{r}}{\rm{e}}{\rm{s}}{\rm{s}}{\rm{e}}{\rm{s}}\,{\rm{t}}{\rm{u}}{\rm{m}}{\rm{o}}{\rm{r}}\,{\rm{g}}{\rm{r}}{\rm{o}}{\rm{w}}{\rm{t}}{\rm{h}}\\ \eta =0 & {\rm{i}}{\rm{f}}\,{\rm{n}}{\rm{o}}\,{\rm{o}}{\rm{A}}{\rm{d}}{\textstyle \mbox{--}}{\rm{M}}{\rm{S}}{\rm{C}}\,{\rm{w}}{\rm{i}}{\rm{t}}{\rm{h}}\,{\rm{i}}{\rm{n}}\,{\rm{t}}{\rm{u}}{\rm{m}}{\rm{o}}{\rm{r}}\,{\rm{s}}{\rm{i}}{\rm{t}}{\rm{e}}\\ \eta  > 0 & {\rm{i}}{\rm{f}}\,{\rm{o}}{\rm{A}}{\rm{d}}{\textstyle \mbox{--}}{\rm{M}}{\rm{S}}{\rm{C}}\,{\rm{p}}{\rm{r}}{\rm{o}}{\rm{m}}{\rm{o}}{\rm{t}}{\rm{e}}{\rm{s}}{\rm{t}}{\rm{u}}{\rm{m}}{\rm{o}}{\rm{r}}\,{\rm{g}}{\rm{r}}{\rm{o}}{\rm{w}}{\rm{t}}{\rm{h}}\end{array}\end{array}$$

and

9 $$\{\begin{array}{c}\begin{array}{cc}{\xi }_{M}=1 & {\rm{i}}{\rm{f}}\,{\rm{o}}{\rm{A}}{\rm{d}}{\textstyle \mbox{--}}{\rm{M}}{\rm{S}}{\rm{C}}{\rm{s}}\,{\rm{a}}{\rm{r}}{\rm{e}}\,{\rm{u}}{\rm{s}}{\rm{e}}{\rm{d}}\,{\rm{a}}{\rm{s}}\,{\rm{o}}{\rm{n}}{\rm{c}}{\rm{o}}{\rm{l}}{\rm{y}}{\rm{t}}{\rm{i}}{\rm{c}}\,{\rm{A}}{\rm{d}}{\rm{s}}\,{\rm{d}}{\rm{e}}{\rm{l}}{\rm{i}}{\rm{v}}{\rm{e}}{\rm{r}}{\rm{y}}\,{\rm{v}}{\rm{e}}{\rm{h}}{\rm{i}}{\rm{c}}{\rm{l}}{\rm{e}}\\ {\xi }_{M}=0 & {\rm{i}}{\rm{f}}\,{\rm{o}}{\rm{A}}{\rm{d}}{\textstyle \mbox{--}}{\rm{M}}{\rm{S}}{\rm{C}}{\rm{s}}\,{\rm{a}}{\rm{r}}{\rm{e}}\,{\rm{n}}{\rm{o}}{\rm{t}}\,{\rm{u}}{\rm{s}}{\rm{e}}{\rm{d}}\,{\rm{a}}{\rm{s}}\,{\rm{o}}{\rm{n}}{\rm{c}}{\rm{o}}{\rm{l}}{\rm{y}}{\rm{t}}{\rm{i}}{\rm{c}}\,{\rm{A}}{\rm{d}}{\rm{s}}\,{\rm{d}}{\rm{e}}{\rm{l}}{\rm{i}}{\rm{v}}{\rm{e}}{\rm{r}}{\rm{y}}\,{\rm{v}}{\rm{e}}{\rm{h}}{\rm{i}}{\rm{c}}{\rm{l}}{\rm{e}}\\ {\xi }_{V}=1 & {\rm{i}}{\rm{f}}\,{\rm{o}}{\rm{n}}{\rm{c}}{\rm{o}}{\rm{l}}{\rm{y}}{\rm{t}}{\rm{i}}{\rm{c}}\,{\rm{A}}{\rm{d}}{\rm{s}}\,{\rm{a}}{\rm{r}}{\rm{e}}\,{\rm{d}}{\rm{i}}{\rm{r}}{\rm{e}}{\rm{c}}{\rm{t}}{\rm{l}}{\rm{y}}\,{\rm{i}}{\rm{n}}{\rm{j}}{\rm{e}}{\rm{c}}{\rm{t}}{\rm{e}}{\rm{d}}\,{\rm{i}}{\rm{n}}{\rm{t}}{\rm{o}}\,{\rm{t}}{\rm{h}}{\rm{e}}\,{\rm{s}}{\rm{y}}{\rm{s}}{\rm{t}}{\rm{e}}{\rm{m}}\\ {\xi }_{V}=0 & {\rm{i}}{\rm{f}}\,{\rm{o}}{\rm{n}}{\rm{c}}{\rm{o}}{\rm{l}}{\rm{y}}{\rm{t}}{\rm{i}}{\rm{c}}\,{\rm{A}}{\rm{d}}{\rm{s}}\,{\rm{a}}{\rm{r}}{\rm{e}}\,{\rm{n}}{\rm{o}}{\rm{t}}\,{\rm{d}}{\rm{i}}{\rm{r}}{\rm{e}}{\rm{c}}{\rm{t}}{\rm{l}}{\rm{y}}\,{\rm{i}}{\rm{n}}{\rm{j}}{\rm{e}}{\rm{c}}{\rm{t}}{\rm{e}}{\rm{d}}\,{\rm{i}}{\rm{n}}{\rm{t}}{\rm{o}}\,{\rm{t}}{\rm{h}}{\rm{e}}\,{\rm{s}}{\rm{y}}{\rm{s}}{\rm{t}}{\rm{e}}{\rm{m}}\end{array}\end{array}$$

The functional form, *D* in Eq. (7), represents a ratio-dependent tumor cell kill by activated CTLs, derived in^38^. The parameters, *α* and *l*, denote the maximum fractional tumor cell lysis by CTLs and a CTL strength scaling exponent, respectively. The parameter *h*_*EC*_ in *D* represents the activated CTL toxicity constant that supports half maximum CTL killing rate. *η* is the tentative tumor growth promotive/suppressive constant induced by oAd-MSCs. Since to our knowledge the number of tumor cells promoted/suppressed by MSCs, *η*, has not been measured experimentally, we chose to focus on the overall MSC promotive/suppressive effect (*δ*_*p*/*s*_*η*), where *δ*_*p*/*s*_ is the probability that an interaction between an MSC and a tumor cell results in promotion/suppression of tumor proliferation. In model simulations, for illustrative purposes, we use a baseline value of *δ*_*p*/*s*_*η* = 0.5 × 4 = 2 cells/day. Thus, to demonstrate the potential confounding mechanisms of promotion/suppression of tumor cell proliferation induced by oAd-MSCs, the simulations are run to ensure that at least 2 tumor cells are suppressed by oAd-MSC. In the case where simulations consider much larger values of *η* than the assumed baseline value, such adjustments will be addressed accordingly in their respective sections. The rest of the model parameters are summarized in Table S1.

Oncolytic Ads have been shown to successfully replicate within and lyse both tumor cells and MSCs^11,12,16^, and for this reason we assume that the cell death response function *l*_*v*_(*MOI*) is a Hill-like function of the multiplicity of infection (MOI). Mathematically, the MOI-dependent kill rate can be formulated as follows:

10 $${l}_{v}(MOI)=\frac{{(MOI)}^{n}}{{h}_{v}^{n}+{(MOI)}^{n}}.$$

The parameters ${h}_{v}^{n}$ and *n* denote the amount of virions (virus particles) necessary to generate half-maximal cell death and the scaling exponent (coefficient) of the Hill function, respectively. The Hill-like function of the multiplicity of infection governs the lysis rate of infected cells by oncolytic Ads which, essentially, depends on MOI (see Appendix B in the SI text). In this study, we emphasize that the higher the MOI, the higher the lysis of the infected tumor cells^18^. We also emphasize that the proposed model does not take into account multiple infection. The Hill function we use in Eq. (10) reflects the effects of an OV on infected cells by accounting for the phenomenon where the lysis, *l*_*v*_, approaches the maximum value (denoted by 1), whenever MOI approaches higher values (i.e., at higher virus loading). Higher values of the exponent *n* > 1 increase the sensitivity of infected cells (oAd-MSCs or tumor cells) to infection of adenovirus. A complete description of the model interactions and the parameter estimates is presented in the SI text.

#### Initial conditions and lysis

Since the model was used to investigate different treatment scenarios, each scenario has a set of initial conditions. We use *in vitro* oAd-MSC data to inform the model parameters on cell lysis of infected MSCs. We assumed the same virus-mediated lysis for tumor cells because the results in^32^ illustrate a MOI (dose)-dependent killing of tumor cells. The cell lysis is, ideally, different in MSC and hepatocellular carcinoma (HCC) cells, since virus replicates more proficiently within HCC. Although we can quantify the oncolytic Ad virus particles released from the MSC as a result of virus replication-mediated cytolysis, it is generally difficult to determine how many virus particles would successfully infect tumor cells. The released virus particles within the hostile tumor microenvironment may be subjected to manifold factors that may hinder their entry into tumor cells^39^. Thus, for simplicity, we assume the same virus-mediated lysis function for both oAd-MSCs and tumor cells. A detailed description of our initial conditions and the parameter estimates is presented in Appendix A in the SI text.

#### Data fitting and parameter estimation

To validate our model, we use an iterated local search-based method which consists of (a) obtaining a local minimum by means of the iterative descent algorithm and (b) randomly changing some of the model parameters and performing another iterative descent algorithm to find a better minimum. The goodness of fit is measured as the residual sum of squares (RSS) between the log of the experimental data and that of the model estimates. The variability among the measured tumor growth curves and response to oncolytic Ad, as well as the oAd-loaded MSC cell viabilities, requires a succinct explanation of data fitting and parameter estimation. We used our model described by Eqs. (1–4) to simultaneously fit the data of Fig. 5(B) of Yoon *et al*.^32^, which corresponds to treatment using oncolytic Adenovirus (oAd) and mesenchymal stem cells (MSC) alone, and a combined treatment of oAd and MSCs. The model fitting was done with the variables *E*_*K*_ and *E*_*C*_ set to zero because the experimental data in^32^ were obtained from immunodeficiency mice which do not mount any immune response to the tumor and/or virus. The experimental data were collected under the following conditions: (i) phosphate buffered saline (PBS) (used as treatment control), (ii) mesenchymal stem cells (MSC), (iii) oncolytic adenovirus (oAd), (iv) MSCs infected with oAd (oAd-MSCs). These cells/viruses were intravenously injected into tumor-bearing mice at 9 and 13 days after the tumor cell injection. Tumor growth was recorded weekly and represents the average of 1–4 orthotopic tumor models. All data sets contain measurements of luciferase-expressing orthotopic Hep 3B tumor sizes indicated by bioluminescence signal intensity obtained as photons acquired per second (p/s) from regions of interest [see^32^, Fig. 5(A,B)]. We fit the submodel solutions of the system described by Eqs. (1–4). For comparing treatment efficacies, treated tumor growth curves (i.e., with either oAd or oAd-MSC) were paired to PBS (regarded as control) and simultaneous fitting was performed for each pair. For the control case (PBS), we fit the logistic submodel defined by Eq. 1, to estimate tumor growth rate, *a*_*T*_, in the absence of treatment. For other tumor-virus related parameters, infection rate of tumor cells (*β*_*T*_), burst sizes of infected tumor cells (*b*_*T*_), and *in vivo* burst size of oAd-MSC (*b*_*M*_), we used the corresponding treated tumor growth data to fit the corresponding submodel given by Eqs. (1–4). Additionally, we estimate the cell death rate (lysis rate) of infected cells using Eq. 9 for a specific MOI and retained this estimate for subsequent model simulations. To further understand the cell viability of oAd-loaded MSCs for all MOIs, we used an appropriate exponential response function (See Supplementary Fig. S6 in the SI text).

## Results

### Model validation and parameter estimation

**Figure 2:**
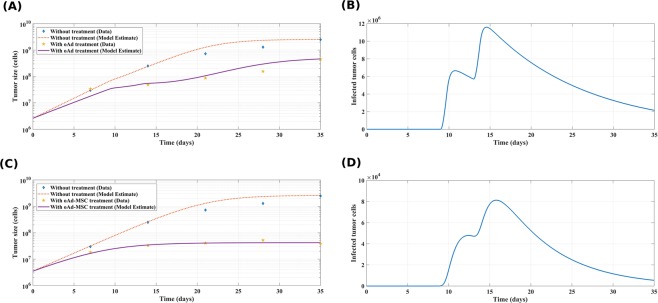
Tumor data and model simulations. Tumor growth data, in the absence of therapy, were fitted to logistic equation **(A)** and to the treatment with oAd-MSC in **(B)**. In **(A**,**C)**, the diamonds represent plots of the untreated tumor (PBS) while the dashed lines denote the model prediction. Similarly, the stars correspond to the oAd-MSC treamtment data while the solutions of the system of Eqs. (1–4) are fitted to the oAd-MSC data with a solid line in **(A,C)**. Our model fits both sets of experimental data well and this fitting enabled robust parameter estimation. In **(B,D)**, a significant tumor reduction is exhibited by a monotonic decrease in size of infected tumor cell population after it reaches a maximum. The oAd-MSC treatment had effectively reduced tumor growth compared either to oAd monotreatment or to the control case (without treatment). These simulations were conducted using the following parameters: (*δ*_*p*/*s*_ = 0), *T*_*u*0_ = 2.6 × 10^6^, *K*_*T*_ = 2.54 × 10^9^, MOI = 5, with other parameters as in Table S1 in SI text.

First, we fit the data corresponding to treatment case (i) with PBS alone. In this case, we used a model that excludes the virus infection and induced tumor/virus specific immune responses. We obtained *a*_*T*_ = 0.315/day and retained this estimated value for all subsequent model simulations. For other remaining tumor-virus related parameters, we sequentially and hierarchically fitted the model parameters to appropriate data sets and obtained the estimates in Table 1 in the SI text. The example plot of both untreated and treated tumor growth data and model solutions fitted to the oAd-MSC treated mice with the measured tumor load *T*_*u*_(*t*) + *T*_*i*_(*t*) is shown in Fig. 2. In Fig. 2 we show representative plots of the model fits to experimental data in^32^. Since in the data it was difficult to determine which cell is infected, the total tumor size (*T*_*u*_(*t*) + *T*_*i*_(*t*)) was measured and the model fitted to the data. The significant reduction of tumor size can clearly be observed by tracking the infected tumor cell population (Fig. 2(B,D)). We note that in the presence of the oAd-MSC, tumor growth is invariably slowed down compared to either oAd treatment or the control case (PBS) at 35 days post tumor implantation, as expected. Consequently, the survival of the oAd-MSC treated mice improves with therapy.

### Predictions and insights from the mathematical model

To better understand the interaction mechanisms of oAd-MSCs in the tumor microenvironment and the effect of MSC-based treatment, we used our new mathematical model to simulate the response of tumor cells to oAd-MSCs. We used our model to provide insights on the optimal use of MSC-based therapeutic strategies for maintaining a lower tumor burden.

The model simulations were based on the following schedule: (1) We first identify the key model parameter using global sensitivity analysis. (2) We simulate the long-term behavior of the model in the absence of therapy (i.e., without the oAd-MSC treatment and the immune response to tumor cells). (3) We then use the computational model to explore various critical properties of MSCs and oncolytic viruses. (4) Finally, we perform an *in silico* therapy to capture the dynamics between the tumor cells, oAd-MSCs, virus populations released from oAd-MSCs and immune cells within the tumor microenvironment. However, due to lack of appropriate data evaluating the tumor promotion or suppression by oAd-MSCs, our mathematical model only presents a conceptual study illustrating the dynamic consequences of local interactions of the tumor-oAd-MSC-immune ecosystem within the tumor microenvironment. Hence, we should be cautious in making certain statements about specific oAd-MSC treatment responses, and any simulation should be interpreted as one *possibility*, and not as an inevitable treatment outcome in any given case. However, a large number of model simulations, along with model sensitivity analysis of parameter fluctuations, can certainly provide a general overview of possible cell-virus dynamics under certain conditions. Further investigations into these mechanisms may generate new insights into the nature of the tumor-oAd-MSC-immune system interactions, as well as effective treatment regimes in oncolytic virotherapy. Next, we highlight four key outcomes from our model simulations.

#### The multiplicity of infection is a key player in tumor reduction and its higher value leads to better treatment outcomes

We first took a more holistic view of the parameter space by performing a global sensitivity analysis using both Pearson Rank Correlation Coefficients (PRCC) and the extended Fourier Amplitude Sensitivity Testing (eFAST). Our sensitivity analysis (see Identification of key model parameters section in SI text) reveals that there is a variability in parameters that influence tumor size at different time points corresponding to early and later stages of tumor growth. Under PRCC, the model system is found to be most sensitive to the multiplicity of infection (MOI) (see Fig. S1 in SI text) as well as to the half-saturation constant that supports half maximum killing of infected cells by the oncolytic virus. Most intriguingly, these parameters can easily be estimated from the experimental data. Furthermore, the eFAST results (see Fig. S2 in SI text), indicate that MOI is the most significant parameter affecting the early stages of tumor growth, while the virus clearance and the rate of CTL recruitment are most significant at latter time points. The qualitative results of our global sensitivity analysis show that the MOI is a key driver of tumor reduction. To further investigate this interesting result and to determine how loading MSCs with different MOI impacts the tumor lysis, we simulated different therapeutic profiles of tumor cell growth by varying MOI values as used in the oAd-MSC as described in our experiments. The resulting relative contributions of each MOI to tumor oncolysis are shown in Fig. S5 in SI text.

We note that lower loading of oncolytic Ads on MSCs does not lead to a decrease in the tumor size, instead the tumor growth is worsened by MSCs, indicating a treatment failure. On the other hand, at higher loading, we notice that not only the tumor is reduced to a small size, but is also rapidly debulked to a small size with the second dose of oAd-MSCs. More importantly, the tumor is kept at small size for longer therapeutic time window. Using this information, we can conclude that loading MSCs at various MOIs, within tolerable toxicities, may improve our current understanding of the therapy adjustments indispensable for successful oAd-MSC based treatment outcomes.

Our sensitivity analysis has significant impacts from both the mathematical and clinical research viewpoints. From the clinical perspective, the sensitivity analysis results suggest that, based on the variations of MOI, tumor cells can be more effectively controlled during the early growth phases (i.e., the oAd-MSC therapy is more likely to be successful during early tumor evolution), while at latter growth phases, the tumor is more likely to escape the therapy (i.e., failure of oAd-MSC therapy is more likely to occur during latter tumor growth phases). This could be due, partly, to high free virus clearance by the anti-virus immune cells (note that the virus clearance is statistically significant at days 70 and 200 - see Fig. S2 in SI text). This particular result is consistent with other models which highlight that increasing virus clearance leads to a larger tumor burden^40^. Taken together, these results elucidate how cell death, which depends on the multiplicity of infection according to this model, can affect clinical outcome. Thus, our sensitivity analysis highlights how parameter space screening to assess the cell death by oncolytic viruses can be useful in designing new oncolytic vectors for MSC-based treatments.

### Anti-tumoral effect of oAd-MSC based therapy depends on their tumor promotive or suppressive action

Considering the fact that it is still currently a contentious debate whether MSCs exert a pro- or anti-tumor action^8^, numerical simulations such as the those conducted herein may aid to objectively predict the consequences of using MSCs for delivering oncolytic Ads *in vivo*. It is known that MSC-based therapies can produce varying outcomes in relation to secretion of cytokines and chemokines, and immunomodulatory potential^24–26^, making it difficult to predict how an individual patient may respond to MSC-cell based oncolytic virotherapy^25^.

Our sensitivity analysis (see Fig. S1 in SI text) indicates that the model is sensitive to immune related parameters at latter times of tumor growth. At the latter growth stages, tumors often express different tumor antigens which are recognized by immune cells, and if on the other hand, the tumor can downregulate, modify or abrogate expression of these antigens, then it may find a way to avoid immune recognition. Our sensitivity analysis results further illustrate that the immune response may not be capable of controlling early tumor growth. This result is also confirmed by the simulations in Fig. S3 in SI text which show how a tumor evolves over time in the presence of the immune response. We therefore, herein examine the impact of oAd-MSCs on tumor growth when there is no immune response. To further investigate the anti-tumoral effect of using oAd-MSCs, we use our mathematical framework to simulate three different treatment protocols when: (a) oAd-MSCs *do not have* any effect on tumor growth; (b) oAd-MSCs *promote* tumor growth; and (c) oAd-MSCs *suppress* tumor growth.

#### (a) oAd-MSCs that do not have any effect on tumor growth offer moderate treatment outcomes

**Figure 3:**
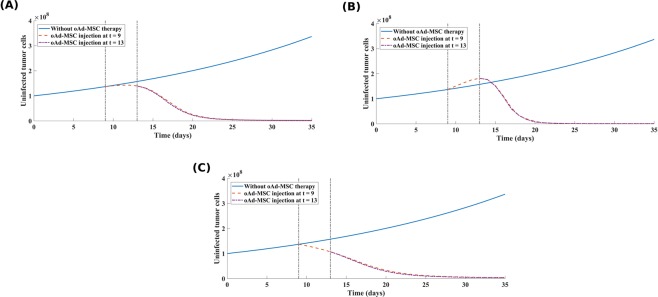
Consequences of oAd-MSC therapy on primary tumor growth dynamics. **(A)** Simulated tumor growth in the case where oAd-MSCs neither promote nor suppress tumor cell proliferation (*δ*_*p*/*s*_ = 0). **(B)** Simulated tumor growth in the case where oAd-MSCs promote tumor cell proliferation, while **(C)** shows simulated tumor growth in the case where oAd-MSCs exert a suppressive effect on tumor cell proliferation. These simulations were conducted using the following parameters: (*δ*_*p*/*s*_ = 0.5), *a*_*T*_ = 0.035^45^, *K*_*T*_ = 5.14 × 10^11^ ^45^, MOI = 5, with other parameters as in Table S1 in SI text.

The simulation results, presented in Fig. 3(A), show that the oAd-MSC treatment exhibits potent killing effects, with cell killing of 99.3% for tumor cells on day 35 suggesting that using oAd-MSCs that have no effect on tumor cell growth can still yield a favourable treatment outcome. We note, compared to cases (B) and (C), that oAd-MSCs that do not have any effect on tumor growth offer better treatment outcomes than oAd-MSCs that suppress tumor growth (case C).

#### (b) oAd-MSCs that promote tumor cell proliferation elicits highly effective anti-tumor responses

The results in Fig. 3(B) indicate that administering oAd-MSCs that promote tumor proliferation (i.e., prior to their lysis by oncolytic virions) effectively exhibited the greatest killing effects, with cell killing of 99.8% for tumor cells on day 35. From both the modeling and clinical perspectives, this result shows that using oAd-MSCs which promote tumor proliferation exhibits higher tumor killing effects is very intriguing, and initially counter-intuitive. Despite the fact that MSCs promote the growth of tumors, infected MSCs (i.e., infected with the replicating virus, such as oncolytic Ad), typically die within 5 days from virus infection^41^. Promising as this observation is, future experimental research is warranted to justify this observation. Results in Fig. 3(B), however, confirm the findings in the experiment that using oAd-MSCs results in effective tumor killing compared to anti-tumor effects of naked oncolytic Ads (see also Table S2 in SI text). Our numerical results are comparable to the findings in^18^ which demonstrated that there was a decrease in tumor burden in mice treated with oncolytic Ad delivered by MSC carriers compared with the direct injection of the oncolytic Ad.

#### (c) oAd-MSCs that suppress tumor growth exhibit a lower tumor killing effect

Finally, we simulated the scenario where the oAd-MSCs exert a suppressive effect on tumor cell growth. Results are depicted in Fig. 3(C). We note that increasing the absolute value of tumor growth promotion/suppression constant in the model only increases the promotive/suppressive effect of oAd-MSC on tumor proliferation, but the model qualitatively demonstrated similar results under the three scenarios considered in this study (see Fig. S4 in SI text). >From Fig. 3(C), we see that if the oAd-MSCs exert some suppressive effect on tumor cell growth, the treatment is significantly less effective than anticipated. Taken together, we conclude that using oAd-MSCs that promote tumor proliferation may provide a significant advantage in tumor cell reduction, though the therapeutic benefit of such oAd-MSCs may be limited if the oncolytic viruses do not successfully infect all tumor cells. Based on these results, if the goal of the therapy is to target long-term tumor control, using oAd-MSCs that have promotive effects on tumor growth offer better treatment outcomes. These results demonstrate that systemic delivery of oncolytic Ad by MSCs may provide a powerful alternative therapy to naked oncolytic Ad. As demonstrated by the simulations, we infer that, because the infected MSCs effectively delivered oncolytic Ads to the tumor site in experiments and in^18^, oAd-MSC based therapy surmounts the limited clinical applicability of system administration of oncolytic Ads and provides effective treatment to inaccessible tumors.

### *In silico* simulations of oAd-MSC based therapeutic dynamics

Given the above observations, we further used our mathematical model to perform *in silico* simulations of oAd-MSC dynamics under two scenarios: High initial oAd-MSC inoculum and comparison of oAd-MSC with direct oAd therapies. The simulation results we present here are purely hypothetical and require further investigations of this tumor treatment. Eventhough the simulation results are interpreted in the context of hypothetical clinical patient outcomes, the simulation settings align with the *in vivo* experiment in^32^, to mimic oAd-MSC based treatment regimes.

#### Increasing oAd-MSCs inoculum leads to a faster reduction of tumor burden

**Figure 4:**
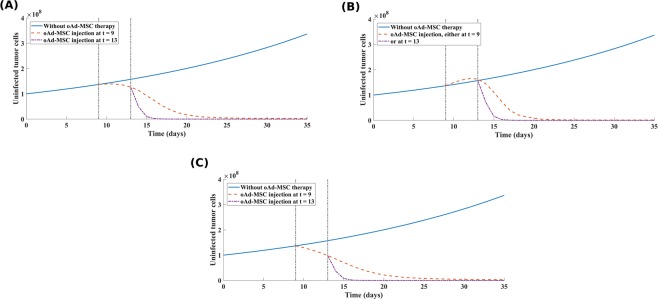
The effect of increasing oAd-MSC dose on tumor cell lysis. A high dosage of 1 × 10^8^ oAd-MSCs is injected into the system on days 9 and 13. The simulated tumor growth in the case where oAd-MSCs have no effect on tumor cell proliferation (*δ*_*p*/*s*_ = 0) is shown in **(A)**. **(B)** Simulated tumor growth in the case where oAd-MSCs promote tumor cell proliferation. **(C)** Simulated tumor growth in the case where oAd-MSCs exert a suppressive effect on tumor cell proliferation. In **(B,C)**, it is assumed that the oAd-MSCs have 50% (*i*.*e*., *δ*_*p*/*s*_ = 0.5) chance of either promoting or suppressing tumor proliferation, respectively. These simulations were conducted using the following parameters: *a*_*T*_ = 0.035^45^, *K*_*T*_ = 5.14 × 10^11^ ^45^, and other parameters as in Table S1 in SI text.

To investigate the dose-dependency of the initial oAd-MSC inoculum, we simulated the same three treatment scenarios discussed above with an increased value of 1 × 10^8^ oAd-MSCs, under the same MOI of 10. While the initial oAd-MSC inoculum can be manipulated in clinical settings, we are predominantly interested in the dynamic interactions that can be observed in the human clinical setting, though it is conceivable that mouse experiments may emulate clinical settings. In any case, the initial oAd-MSC inoculum injected into patients would be higher than those considered in animal models^24^. Note that in our simulations here, we used the same number of oncolytic Ads (5 × 10^8^ viral particles (VP)) as described in our experiments above, and increased number of MSCs (1 × 10^8^ cells), generating MOI of 5, which is still within the clinical ranges reported in^24^. The results of this adjustment are shown in Fig. 4.

From Fig. 4(A), we see that the second dosage at day 13 of oAd-MSCs is able to drastically reduce the tumor size by 99.84% compared to what is in Fig. 3(A). It is interesting to note that although the oAd-MSCs injected do not impact a tumor in anyway, the progeny of oncolytic Ads released from oAd-MSCs is able to effectively reduce tumor to small sizes. Again, comparing Figs. 3(B) and 4(B), where oAd-MSCs promote tumor proliferation, we observe that the high dosage of oAd-MSCs is able to greatly reduce the tumor by approximately 99.87%. Finally, we also observe an enhanced tumor killing of approximately 100% in Fig. 4(C) compared to 99.9% in Fig. 3(C). For clinical applicability we adapted a high infusion of 1 × 10^8^ oAd-MSCs (which is less than 3.0 × 10^8^ cells (clinical dose)^41^), and we noted that in Fig. 4, the second oAd-MSC dosage is capable of rapidly reducing tumors to small sizes compared to Fig. 3. This implies that tumors could be held in a small controllable state by the presence of oAd-MSCs, within the tumor microenvironment, which would ultimately produce a second progeny of Ads that can propagate and infect more tumor cells.

#### oAd-MSC therapy provides better therapeutic outcomes compared to direct oncolytic Ad therapy

**Figure 5:**
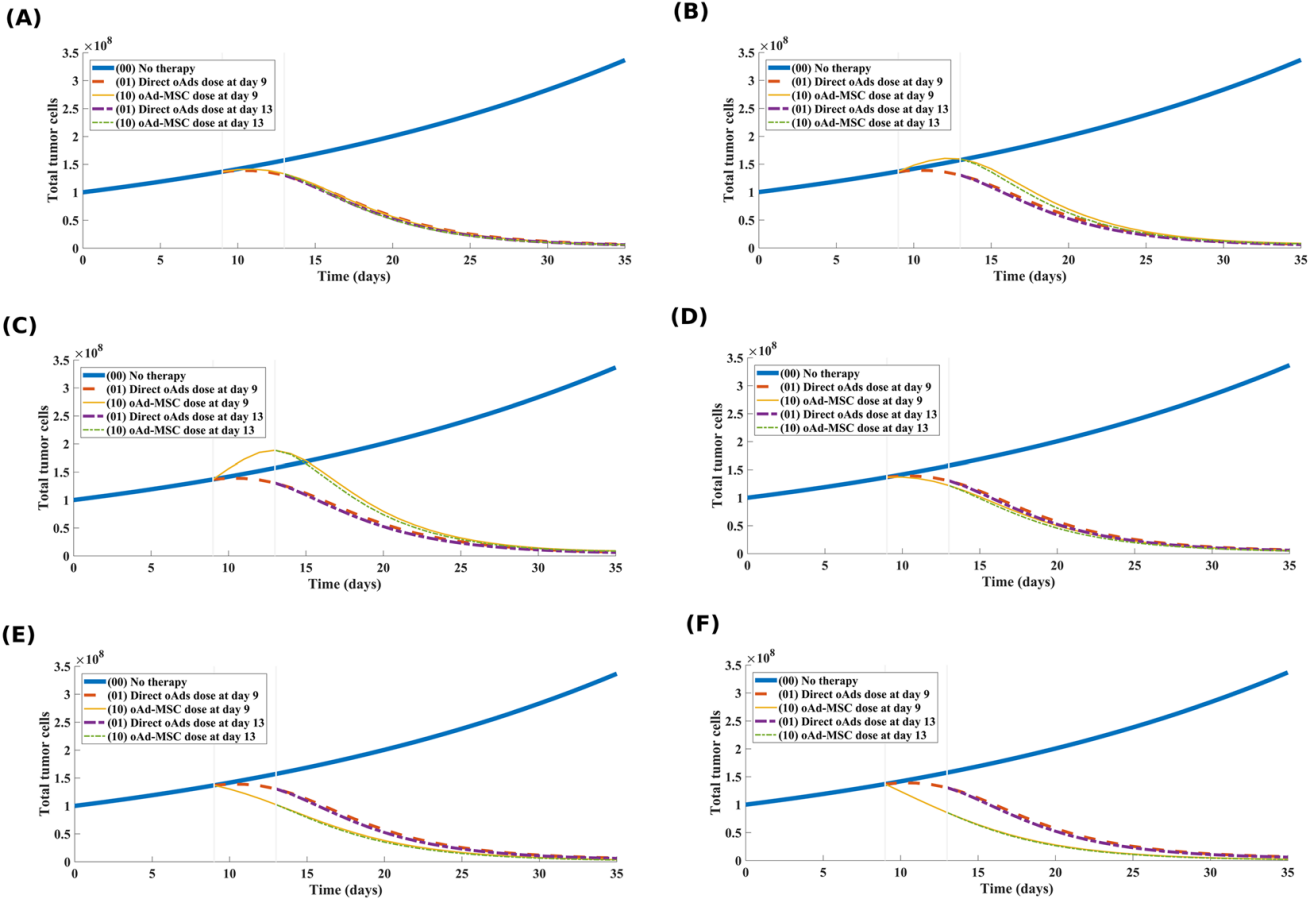
Comparison of direct oncolytic Ads dose versus oAd-MSC dose. Comparison of direct oncolytic Ads dose with the dose of oAd-MSCs that promote tumor growth (**(A)** by 10%(*δ*_*p*/*s*_ = 0.1), **(B)** by 50%(*δ*_*p*/*s*_ = 0.5), and **(C)** by 90%(*δ*_*p*/*s*_ = 0.9)), and with oAd-MSCs that suppress tumor growth (**(D)** by 10%(*δ*_*p*/*s*_ = 0.1), **(E)** by 50%(*δ*_*p*/*s*_ = 0.5), and **(F)** by 90%(*δ*_*p*/*s*_ = 0.9)). Note the following notation for treatment scenarios: No therapy (00), direct injection of naked oncolytic Ads only (01), injection of oAd-MSCs only (10). The low promotive/suppressive probability (*δ*_*p*/*s*_ = 0.1) in **(A,D)** indicates negligible advantage of oAd-MSC therapy over the therapy naked oncolytic Ads. Also note that at higher probability (*δ*_*p*/*s*_ = 0.9), in **(C,F)**, the oAd-MSC therapy reduces tumor burden faster than the naked oncolytic Ads. This indicates that if the oAd-MSCs have a high chance of promoting/suppressing tumor cell proliferation, then the oAd-MSC therapy provides better therapeutic outcomes, as compared with direct intravenous dose of naked oncolytic Ads.

Despite a growing need for the best therapeutic options, clinical goals are now turning towards optimization of long-term control of cancer, rather than a complete cure. Mathematical models are useful tools for enhancing our understanding of the optimal therapeutic options. In this regard, we chose to test the merit of oAd-MSC regimens by comparing them with direct intravenous injection of naked oncolytic Ads. In particular, we were interested in finding which therapy regimen leads to rapid tumor reduction. We assume that a treatment regimen that reduces a total tumor cell population rapidly, within the therapeutic window of 35 days as in the experiments herein, will offer a greater tumor control, minimize time to tumor relapse, and provide a better prognosis. In order to compare the significance of each therapy, dependent on the influx terms (described in model Eqs. (3) and (4) which have switch boolean constants (*ξ*_*M*_ and *ξ*_*V*_) defined in Eq. (9) in SI text), we held one term constant and vary the other according to the following therapy schedules: (1) no therapy; (2) direct injection of naked oncolytic Ads only; and (3) injection of oAd-MSCs. Note also that these treatment schedules are simulated in consideration of whether oAd-MSCs promote or suppress tumor cell proliferation and for different probabilities of tumor growth promotion/suppression by oAd-MSCs. The results are shown in Fig. 5.

In Fig. 5 (see also Table S2 in SI text), we note that when oAd-MSCs have a smaller likelihood of promoting or suppressing tumor cell growth, the oAd-MSC based regimen yields a relatively similar treatment outcome to direct dose of naked oncolytic Ads. Most importantly, when oAd-MSCs have a higher likelihood of suppressing tumor cell proliferation, our results (Fig. 5(F)) indicate that the oAd-MSC therapy outperforms the direct dose of naked oncolytic Ad therapy. In general, this result indicates that the oAd-MSC treatment is not only more effective at controlling tumor burden, but also reduces tumor size rapidly, suggesting that the oAd-MSC treatment has important therapeutic outcomes over the therapy with naked oncolytic Ads.

In summary, the simulations conducted in this study shed light on different plausible treatment outcomes which may arise when using MSCs as cellular vehicles of oncolytic viruses. Importantly, though the above simulations were done under hypothetical clinical settings, the simulation results are qualitatively comparable to the clinical results in^42^. For example, in^42^ a complete clinical response was observed in four children when MSCs were used as cellular carriers of oncolytic virus, and one of the four children was reported to have complete remission 3 years after MSC-based oncolytic virotherapy. Taken together, our computational framework described herein may serve as a basic platform which can help to objectively assess the contribution of oAd-MSC based dynamics to clinical outcomes, as it is often difficult to predict how individual patients may respond to the MSC-based carrier therapies^25^.

## Discussion and Conclusion

In this paper, we devised a new mathematical model calibrated with a set of experimental data, including data from our own *in vitro* and *in vivo* experiments^32^, for the delivery of oncolytic Ads by MSCs. Even though mathematical models that describe the use of cells as carriers of therapeutic agents to tumor sites have been proposed in the literature (see for example^30,31^), no model has described the dynamics of tumor growth under MSC-based oncolytic virotherapy. Our quantitative framework is the first of its kind in that it not only describes the local interactions of tumor cells with oncolytic viruses and immune cells, but also incorporates the impact of MSC promotive/suppressive actions on tumor growth dynamics.

We quantified how model parameters influence tumor growth dynamics with global sensitivity analysis of the parameter space. The analysis revealed that the lysis rate of infected cells, which depends on the multiplicity of infection (MOI), is the most influential parameter. In particular, low variation in MOI suggests that the oncolytic Ads have a great potential of destroying tumor cells at all time points. This is qualitatively affirmed by the model simulations, especially in Fig. 4. In our fitting, we kept the multiplicity of infection (MOI = 5) constant because the MOI of 5 led to the optimal condition for viral production in^32^. However, comparison of lysis rates for tumors treated with direct oAd with those treated with oAd-MSC suggests that the initial viral dose is an important variable and a key determinant of successful therapy. While higher initial viral dose may favor treatment with direct oncolytic virus, higher virus inoculum may critically induce premature MSC lysis (due to adenovirus replication), leading to decreased overall efficacy of oAd-MSC therapy^32^. We performed a global sensitivity analysis of the parameter space and model simulations with the current model and noticed that this is true. Experimental evidence from various tumor models with oAd support this conclusion^11,32,43^.

Based on this understanding, it seems that oAd-MSCs can significantly enhance oncolytic virotherapy. This may, at least in part, offer reasonable justification for why the use of MSCs as cellular vehicles of OVs appears to be the most attractive therapy in anti-tumor strategies. Altogether, our PRCC and eFAST results reveal that the lysis rate of infected cells is related to the enhanced oAd-MSC treatment efficacy, consistent with the experimental findings in^16,17^. We should emphasize that variation in lysis rate of infected cells could be more accurately quantified given the availability of relevant patient-specific data, and the efficacy of oAd-MSCs as cellular vehicles is more likely to influence clinical outcomes. Therefore, quantifying MOI variations could provide a significant impact on the design of future oAd-MSC based therapies that target tumor cell proliferation.

We further used the model to investigate how systemic administration of oAd-MSC influences the treatment outcomes compared to direct administration of naked oncolytic Ads. In particular, we were predominantly interested in finding out how the dynamic interactions between oAd-MSCs and tumor cells impact the therapeutic outcome, under the notion that the Ad-MSCs may promote/suppress tumor proliferation. Given the low infusion of oAd-MSCs (Fig. 3(B)), we note that, though the tumor is effectively reduced, there is still a large number of uninfected tumor cells at the end of the experimental therapeutic window of 35 days. This offers a slight improvement compared to the case where oAd-MSC have no influence on tumor growth (Fig. 3(A)). If, on the other hand, the oncolytic Ads cannot successfully infect a large portion of tumor cells, then this result suggests that oAd-MSCs could allow tumors to escape therapeutic control. This is in line with anti-tumoral studies indicating that MSCs promote tumor progression and metastasis in animal models^8,44^.

We alternatively simulated the scenario where oAd-MSCs suppress tumor growth (Fig. 3(C)), under low infusion of oAd-MSCs. We observed that the tumor is not greatly reduced as anticipated, compared to the previous scenario indicated in Fig. 3(B). This scenario is, however, still comparatively better than the case where oAd-MSCs exert no effect on tumor growth (Fig. 3(A)). This result suggests, at least from the clinical safety control perspective, that using oAd-MSCs that suppress tumor growth could still offer improved anti-tumor treatment outcomes. It is important to note that our model captures dynamical behaviour across a range of treatment scenarios (three scenarios considered here). While each scenario is described by a specific choice of parameters, there is an intriguing underlying behaviour common to all scenarios. In particular, our model simulations explicitly display exponential tumor growth, indicating unbounded early tumor growth dynamics, and population regression following the administration of oAd-MSC therapy, suggesting the feasibility and efficacy of using oAd-MSCs. In general, our results indicate that oAd-MSCs are capable of interacting with tumor cells in various ways which consequently lead to a reduced tumor cell population, consistent with both experimental and clinical studies^18,24,42^.

We also compared the therapeutic outcomes of injecting high oAd-MSC inoculum (emulating clinical settings, similar to settings in^24^) to low dosage of oAd-MSC (emulating sub-clinical or experimental animal settings) on tumor reduction. The results of high infusion of 1 × 10^8^ oAd-MSCs are shown in Fig. 4, while results of injecting a small dose of 1 × 10^6^ oAd-MSCs are indicated in Fig. 3. Investigation of tumor reduction revealed that high injection of oAd-MSCs causes, not only a faster reduction in tumor burden, but a massive reduction of the tumor under all three treatment scenarios. These results show a dose-dependent increase in tumor killing efficacy of oAd-MSC therapy, indicating that oncolytic Ads (delivered by MSCs) are capable of replicating efficiently and inducing tumor cell death. Based on this understanding, we conclude that treatment with high infusion of oAd-MSCs provides a more useful tool compared with treatment with low injection of oAd-MSCs, consistent with the findings in^24^. It is crucial to note that even though we simulated therapeutic scenarios with high injection of oAd-MSCs, the cell viability of MSC post-infection decreases rapidly as shown in^17^, in particular, infected MSCs typically die within 5 days from virus infection as confirmed in^41^.

Having confirmed that the initial inoculum of oAd-MSCs plays a critical role in tumor control, we were interested in modeling how the MOI-dependency of oAd-MSC impacts treatment outcome. We showed that low MOIs may lead to treatment failure, while higher values of MOIs yield better results than previously thought, with tumor significantly reduced and maintained in small sizes (see Fig. 4). As demonstrated above, this finding highlights the need to find the right balance between the number of oAd-MSCs injected and the amount of oncolytic virions loaded on the MSCs for long term tumor control with OVs. The appropriate MOI adjustments can help determine the tumor size outcome with, possibly, a tumor-free state at the end of therapy. When comparing the therapeutic benefits of using oAd-MSC therapy to direct intravenous dose of naked oncolytic Ads, our simulation results (Fig. 5) point out that the oAd-MSC therapy offers qualitatively similar, but slightly better, prognosis with respect to rapid reduction of tumor cells, than the naked oncolytic Ad therapy. Thus, our results highlight an essential subtlety that can further be tested in future clinical trials focused on this comparison.

Our computational analysis provides new important insights into the properties of MSCs when used as cellular vehicles for delivering oncolytic Ads to the tumor microenvironment. Understanding the dynamics of how tumors respond to oAd-MSC therapies can inform oncolytic virotherapy schedules and can provide basic guidelines for optimizing treatment response. Even though the results of this study are novel and promising, several limitations exist in the current modeling framework. With respect to the lysis term proposed here, model predictions may change as a function of multiplicity of infection (MOI) and the Hill coefficient (*n*), as confirmed by global sensitivity analysis results in Figs. S2 and S3. Even with these limitations, the lysis term proposed in this modeling approach allowed for the prediction of tumor response to oncolytic Ads delivered by MSCs, as observed in the oAd-MSC experiments we carried out. While no violation of model assumptions is observed, it is inherently difficult to test whether the antiviral immune response and anti-tumor immune response in the model are independently induced by OVs, as the experimental data in our study were derived from human xenograft tumor-bearing immunodeficient mice. Thus, it should be noted that the model predictions cannot be applied directly to the human clinical setting, and there is a need for robust model calibration and validation as appropriate data become available.

Most of the parameters pertaining to the interaction of tumor cells, immune cells, and OVs used in the current model were obtained from previous models, which fitted models to experimental data from immunodeficient mice, due to lack equivalent data for human patients. We, therefore, emphasize that parameter re-evaluation is certainly warranted in future to perform rigorous model validation. While also conceivable that model predictions may vary, the fundamental concepts relating to tumor growth promotion/suppression induced by MSCs are robust to parameter choice, and qualitatively similar results are expected to be observed. Importantly, optimization of the oAd-MSC dosing regimens and incorporation of pharmacokinetic/pharmacodynamic terms describing the release of cytokines/chemokines by MSCs to promote/suppress tumor growth would be an attractive further extension of the current model.The presence of tumor growth promotive/suppressive cytokines/chemokines within the tumor microenvironment could strongly influence the outcome of the local interactions of tumor cells with oAd-MSCs, immune cells, and OVs.

In conclusion, this study illustrates different tumor responses which are difficult to explain from the experimental results alone. Specifically, this model provides a new theoretical approach for predicting tumor changes in response to oncolytic Ads *in vivo* delivered by MSCs. Despite the inherent simplifying assumptions in the proposed model, our computational approach may serve as a basic platform for further refinement of mathematical models that describe tumor responses to cell-based virotherapies. Our model may, at least in part, offer justification to why oAd-MSCs should be used with great caution under clinical settings. Most importantly, our results highlight that oAd-MSCs provide a feasible synergism of oncolytic virotherapy when used as cellular delivery vehicles. Currently, clinical trials using MSCs as cellular vehicles for delivering OVs are under way and our mathematical framework adds new valuable information which may help to determine how MSCs may actually translate into meaningful clinical outcomes. Taken together, the findings in the present paper suggest oAd-MSC therapy as a promising therapeutic candidate for delivering oncolytic Ads for future clinical trials against aggressive and inaccessible tumors.

## Supplementary information

Supplementary Information.

## Data Availability

All data used in this study are available within the article and its supplementary information files.
