# Supplementary material for: Mesenchymal stem cells used as carrier cells of oncolytic adenovirus results in enhanced oncolytic virotherapy
Source: Sci Rep. 2020 Jan 16;10:425. doi: 10.1038/s41598-019-57240-x (PMC6965634; doi:10.1038/s41598-019-57240-x)
Supplement: Supplementary file 1 — Supplementary Information. [file 41598_2019_57240_MOESM1_ESM.pdf]

Mesenchymal stem cells used as carrier cells of oncolytic adenovirus results in enhanced oncolytic virotherapy

Supplementary Information

Khaphetsi Joseph Mahasa[a,b,1], **Lisette de Pillis**[c], **Rachid Ouifki**[b], **Amina Eladdadi**[d], **Philip Maini**[e], **A-Rum Yoon**[f], **and Chae-Ok Yun**[f, 2]

[a]DST/NRF Centre of Excellence in Epidemiological Modelling and Analysis (SACEMA), University of Stellenbosch, Stellenbosch, South Africa
[b]Department of Mathematics and Applied Mathematics,University of Pretoria, Pretoria, South Africa
[c]Department of Mathematics, Harvey Mudd College, Claremont, CA, USA
[d]Department of Mathematics, The College of Saint Rose, Albany, NY, USA
[e]Wolfson Centre for Mathematical Biology, Mathematical Institute, University of Oxford, United Kingdom
[f]Department of Bioengineering, College of Engineering, Hanyang University, Seoul, Republic of Korea
[1,2]Corresponding Authors: mahasa@aims.ac.za & chaeok@hanyang.ac.kr

ABSTRACT

Mesenchymal stem cells (MSCs) loaded with oncolytic viruses are presently being investigated as a new modality of advanced/metastatic tumors treatment and enhancement of virotherapy. MSCs can, however, either promote or suppress tumor growth. To address the critical question of how MSCs loaded with oncolytic viruses affect virotherapy outcomes and tumor growth patterns in a tumor microenvironment, we developed and analyzed an integrated mathematical-experimental model. We used the model to describe both the growth dynamics in our experiments of firefly luciferase-expressing Hep3B tumor xenografts and the effects of the immune response during the MSCs-based virotherapy. We further employed it to explore the conceptual clinical feasibility, particularly, in evaluating the relative significance of potential immune promotive/suppressive mechanisms induced by MSCs loaded with oncolytic viruses. We were able to delineate conditions which may significantly contribute to the success or failure of MSC-based virotherapy as well as generate new hypotheses. In fact, one of the most impactful outcomes shown by this investigation, not inferred from the experiments alone, was the initially counter-intuitive fact that using tumor-promoting MSCs as carriers is not only helpful but necessary in achieving tumor control. Considering the fact that it is still currently a controversial debate whether MSCs exert a pro- or anti-tumor action, mathematical models such as this one help to quantitatively predict the consequences of using MSCs for delivering virotherapeutic agents in vivo. Taken together, our results show that MSC-mediated systemic delivery of oncolytic viruses is a promising strategy for achieving synergistic anti-tumor efficacy with improved safety profiles.

Contents

Description of the model interactions 2
Table of model parameters 3
Identification of key model parameters: Global sensitivity analysis results 3
Tumor growth in the absence of therapy and in the presence of strong immune response 5
oAd-MSC promotive/suppressive-associated results 5
Multiplicity of infection (MOI)-associated results 6
Comparison of oncolytic Ad-based therapies-associated results 6
Appendix A: Initial conditions and parameter estimates 6
Appendix B: Tumor evolution in the absence of therapy but in the presence of the immune response 10
Appendix C: Tumor evolution in both the presence of OVs (without MSCs as delivery vehicle) and the immune response 10
References 12

Description of the model interactions

In equation 1, the first term, $a_T\left(1+\frac{\delta_{p/s}\eta M_i}{h_{TM}+M_i}\right)T_u\left(1-\frac{T_u+T_i}{K_T}\right)$, indicates that in the absence of immune response, the uninfected tumor cells grow logistically with an intrinsic and/ or oAd-MSC-induced growth rates, a_T and $a_T\eta$, respectively. Of note, prior to virotherapy (i.e., in the absence of oAd-MSCs within the tumor microenvironment, $\eta = 0$ in Eq.(8)), uninfected tumor cells are modeled to have a constant proliferation rate, a_T. In the absence of unequivocal data regarding the rate of tumor growth promotion or suppression by an MSC, for simplicity, we assume that the rate of tumor growth promotion is the same as the rate of tumor growth suppression by the MSC. Thus, the coefficient $\delta_{p/s}$ is the probability that local interactions between the uninfected tumor cell and an MSC causes the tumor cell to grow or become suppressed. The parameter K_T denotes the tumor carrying capacity. The parameter h_{TM} is the half-saturation constant for tumor cells that supports half the maximum interaction with an MSC (leading to tumor growth or tumor suppression). The second term, $-\beta_T(t_i)T_uV$, denotes infection of tumor cells by oncolytic virions (V) released at time t_i within the tumor microenvironment. Note that the infection rate, $\beta_T(t_i)$, depends on the time of infection t_i (i.e., the time when the oAd-MSCs arrive and, possibly, release the Adenovirus (Ad) within the tumor microenvironment). Also notice that $\beta_T(t_i) = 0$ for $[0,t_i)$, and $\beta_T(t_i) > 0$ for $[t_i,t_i^*]$, where t_i^* is the terminal time of the experiment. The third term, $-\lambda_T E_K T_u$, represents a direct NK-induced tumor cell death, with the rate of tumor cell death λ_T. This multiplication response term is usually employed when one assumes a continuous killing rate of both uninfected and infected tumor cells by the innate immune system, mediated by NK cells. For simplicity, we only assumed mass-action interaction kinetics between tumor and NK cells. This simplified interaction term has successfully been used to describe the depletion of tumor cells by NK cells during oncolytic virotherapy[1]. The last term, $-DT_u$, represents tumor cell lysis by activated tumor-specific CTLs. The term D, defined in Eq.(7), represents a ratio-dependent CTL-induced tumor cell death. The Holling response function for CTL, in Eq.(7), is usually employed when one assumes that the recruitment of CTLs occurs outside the tumor microenvironment. Normally, T cells are activated to become CTLs in the spleen and traffic to tumor bed to mount their antitumor attack. This function form of cell lysis is a novel term derived by de Pillis et al.[2]. More information and justification of this ratio cell lysis can be found in[2] and[3]. This term has successfully been employed in a number of models[4,5].

In equation 2, an instantaneous transfer of a population of uninfected tumor cells to infected cell population is represented by the first term, $\beta_T(t_i)T_uV$. The oncolytic lysis of infected tumor cells is denoted by the second term, $-l_v(t_i,MOI)T_i$, with lysis rate $l_v(t_i,MOI)$. The lysis rate, $l_v(t_i,MOI)$, depends on the time of infection, t_i, and the multiplicity of infection, MOI. We assume that the death of the infected cells occurs very rapidly following the viral infection; hence, the intrinsic growth of infected cells is neglected. The oncolytic viral infections often foster infected tumor cells to express tumor antigens which are recognised by NK cells[6,7]. Hence, the rate at which NK cells lyse infected tumor cells is represented by the third term, $-\lambda_T E_K T_i$, where λ_T is the rate of NK-induced tumor death. The last term, DT_i, denotes the tumor cell death induced by CTLs (assumed to be similar the cell lysis of uninfected tumor cells by activated CTLs).

In equation 3, the first term, $\xi_M u_M(t)$, represents a pulse infusion of oAd-MSCs into the tumor microenvironment, where ξ_M is a "switch" boolean constant defined in Eq (9). In the experiment described above, the oAd-MSCs were intravenously injected on days 9 and 13 post-implantation of 1×10^6 firefly luciferase-expressing Hep3B cells into the left lobe of the liver in athymic nude mice. Mathematically, we represent these oAd-MSC injections as $u_M(t) = u_{M0}(\delta(t-9)+\delta(t-13))$, where $u_{M0} = 1\times10^6$ cells, $\xi_M = 1$ or $\xi_M = 0$ if oAd-MSCs are used as delivery vehicles or not as delivery vehicles, respectively. Thus, here $u_M(t)$ is the rate at which new oAd-MSCs are injected into the tumor microenvironment at time t and $\delta(t)$ is the Dirac delta function. The last term, $-l_v(t_i,MOI)M_i$, represents lysis of MSC carrier cells within the tumor microenvironment by the pre-loaded replication-competent oncolytic Ad.

In equation 4, $\xi_V u_V(t)$, denotes a pulse intravenous injection of oncolytic Ads into the system, where ξ_V is a "switch" boolean constant defined in Eq (9). In the experiment described above, the oncolytic Ads were intravenously injected on days 9 and 13 post-implantation of 1×10^6 firefly luciferase-expressing Hep3B cells into the left lobe of the liver in athymic nude mice. We represent these oncolytic Ads injections as $u_M(t) = u_{V0}(\delta(t-9)+\delta(t-13))$, where $u_{V0} = 5\times10^8$ virus particles (VP) of oncolytic Ad. Note that $\xi_V = 1$ if oncolytic Ads are directly injected into the system (e.g., oncolytic Ads were directly into the mice in the experiment above) or $\xi_V = 0$ if not directly injected into the system. The term $l_v(t_i,MOI)b_M M_i$ represents the production of new virions from the lysed oAd-MSCs, where $l_v(t_i,MOI)$ is the lysing rate of an MSC carrier. During oncolytic virus propagation (or upon lysis) within an infected cell, new infectious virions are released from each infected cell. Thus, b_M is the burst size for viruses from the MSC carriers. The release virons can, immediately, infect tumor cells in the vicinity of the release point. After successful virus replication within infected tumor cells, T_i, new virus particles are further released and continue to infect the neighbouring uninfected tumor cells. Hence, the term $l_v(t_i,MOI)b_T T_i$ represents the production of new virions from the lysed infected tumor cells, with the lysis rate $l_v(t_i,MOI)$, and the burst size b_T. An immune induced

virus inactivation and elimination is represented by the last term, ωV, where ω is the virus clearance rate within the tumor microenvironment. Note that in the tumor microenvironment, free viruses are susceptible to neutralization by circulating antibodies and/or other antiviral immune cells.

As part of the innate immunity, NK cells are always present at the tumor site[8], and have been shown to play a vital role in immunosurveillance of tumors[9]. Thus, in **equation** 5, the first term, $S_{E_K}(t)$, represents a constant supply of NK cells into the tumor microevironment, as in previous models[2,10]. The second term $-r_K \lambda_T E_K (T_u + T_i)$ represents the inactivation of NK cells as a result of their interaction with tumor cells. The proportion of NK cells that are inactivated during tumor-NK cell interactions is represented by r_K, and λ_T is the rate of NK-induced tumor death. Note that this inactivation occurs when an NK cell encounters a tumor cell several times and consequently ceases to be cytotoxic, and undergoes apoptosis[8]. This mass-action term has successfully been employed in previous models of tumor-immune interactions[2,4,10,11]. The natural death of NK cells is represented by the last term, $-\mu_K E_K$, where μ_K is the rate of NK cell death.

In equation 6, the CTL recruitment to the tumor microenvironment occurs due to the presence of tumor antigens or oncolytic cell death that often exposes a plethora of tumor associated antigens. This antigenic recruitment is denoted by the first term, $-\gamma E_C$, where γ is the recruitment rate of CTLs. Note that this term is negative because CTLs are present at the tumor site only when tumor cells are present, as in[2,10]. The second term $-r_C E_C (T_u + T_i)$ represents CTL inactivation, at the rate r_C, as a result of their interaction with tumor cells. The last term, $-\mu_C E_C$, represents the natural death of CTLs, with the constant death rate μ_C.

Table of model parameters: The baseline parameter values for our model are given in Table S1.

Identification of key model parameters: Global sensitivity analysis results

In this study, most of the parameters have been sourced from the literature. There were, however, some parameters which could not be obtained from the available literature due to lack of appropriate studies or experimental data. We performed two most reliable global sensitivity analyses to assess how model outputs depend on all model parameters. In this way, we hope to determine which model parameters are most influential on tumor cell population ($T_{tumor}(t) = T_u(t) + T_i(t)$) at days $9, 13, 70$ and 200. Note that the first two time points correspond to the oAd-MSC injections as described in the experiments, and the last two are *ad hoc* values which were chosen only for exploring the model sensitivity at later time points. The implemented methods of sensitivity analyses were: the Pearson Rank Correlation Coefficients (PRCC) and extended Fourier Amplitude Sensitivity Testing (eFAST)[18,19]. PRCC is considered to be the most effective method when there is a nonlinear but monotonic relationship between model inputs (or parameters) and outputs (or variables)[20], while eFAST, a variance based method, is considered to be the most reliable method whenever the relationship between the model inputs and outputs is nonlinear and non-monotonic[21]. Even though each method can effectively be used individually, for a complete global sensitivity analysis, it is often recommended to use both methods to capture any nonlinear relationships between model inputs and outputs[18].

The Pearson Rank Correlation Coefficients (PRCC). We generated 1000 sample values for each parameter from a uniform distribution. When sampling all the model parameters, we chose the range of each parameter to vary from $1/2$ to twice its baseline values listed in Table S1 in the Supplementary Material, except $\delta_{p/s}$ which is chosen from 0 and 1. In this choice of parameter ranges, we allow the PRCC to take account of potentially large uncertainty in the model parameters. We then used a Latin Hypercube sampling (LHS) method proposed in[18], along with PRCC values and corresponding p-values, to assess the sensitivity of the model output of interest (tumor cell population) to each parameter at the given time points (i.e., at days $9, 13, 70$ and 200).

Note that the PRCC varies between 0 and 1. Here, the PRCC results for the tumor cell population are interpreted as follows: (1) a negative PRCC value (indicating a negative correlation) with p-value smaller than 0.01 means that increasing the value of that parameter under consideration will decrease the total tumor cell population and hence increase the (relative) efficacy of the oAd-MSCs. (2) A positive PRCC with p-value smaller than 0.01 bears the counter-intuitive meaning of the negative PRCC value, that is, it will diminish the efficacy of the oAd-MSCs. Note that the parameters with large absolute PRCC values greater than 0.1 (i.e., PRCC $> |0.1|$), with corresponding small p-values < 0.05, are regarded as the most influential parameters in the model[22].

As depicted in Fig. 1, our sensitivity analysis reveals that parameter sensitivity varies with the growing tumor. For instance, tumor cell population is highly sensitive to the relatively small changes in the tumor cell proliferation rate, a_T, in the early phases of the tumor growth, but becomes less and less sensitive as the tumor grows. On the other hand, the tumor cell population is sensitive to the lysis rate, as revealed by the influence of the multiplicity of infection, MOI, half-saturation constant that yields half maximum killing of tumor cells by the oncolytic virus, h_v^n, and the Hill function coefficient, n, at all time points. This particular result is consistent with the findings in[23], where chimeric antigen receptor–engineered T cells when used as oncolytic virus carriers. The results are further in agreement with the experimental findings in[24,25] (e.g, see fig. 1A in[24,25]) which showed that there was an increase in the numbers of infected cells with corresponding increase in MOI.

Table S1. Baseline parameter values used in the model simulations and sensitivity analysis. Estimates from the experimenatal data are taken at MOI 10, day 5 post-infection of MSCs.

Parameter	Description	Value	Source
a_T	Intrinsic tumor growth rate	0.315 day^{-1}	Estimate
$\delta_{p/s}$	Prob of promotive or suppressive interaction between tumor cells and oAd-MSCs	0.5	Estimate
η	Tumor growth promotive/suppressive constant by oAd-MSCs	4 day^{-1}	Estimate
h_{TM}	Half-saturation constant that supports half-maximum tumor growth or suppression	500	Estimate
K_T	Tumor carrying capacity	1.47×10^{12} cells	[12, 13]
ξ_M	"Switch" parameter for oAd-MSC therapy	0 or 1 dimensionless	Estimate
ξ_V	"Switch" parameter for direct oncolytic Ad therapy	0 or 1 dimensionless	Estimate
β_T	Infection rate of tumor cells	12.8×10^{-4} virion^{-1} day^{-1}	Estimate
λ_T	Rate of NK-induced tumor death	8.68×10^{-10} cell^{-1} day^{-1}	[14]
α	Maximum proportional tumor kill by CTLs	$\frac{7}{20}$ day^{-1}	[4]
l	Immune strength scaling exponent	$\frac{2}{3}$ dimensionless	[4]
n	Scaling exponent in function l_v which defines cell lysis by oncolytic Ad virions	2 dimensionless	Estimate
h_v	Half-saturation constant that supports half-maximum cell lysis by Ad virions	20	Estimate
h_{E_C}	Activated CTL toxicity constant that supports half maximum CTL killing rate	1.4	[4]
l_v	Rate of death by lysis (MOI=10)	2.0×10^{-1} day^{-1}	Estimate
b_M	Number of virions released from the MSC carriers	100	Estimate
b_T	Number of virions released from an infected tumor cell	1000	Estimate
ω	Virus clearance rate	2.3 day^{-1}	[15]
S_{E_K}	Constant external source of NK cells	1.30×10^{4} cells·day^{-1}	[3]
r_K	Fraction of inactivated NK cells during NK-tumor interactions	1.0×10^{-7} cell^{-1} day^{-1}	[3]
μ_K	Natural death rate of NK cells	4.12×10^{-2} day^{-1}	[3, 10, 16]
γ	Recruitment rate of CTLs	9.0×10^{-3} day^{-1}	[5, 17]
r_C	Fraction of inactivated CTLs during CTL-tumor interactions	3.42×10^{-10} cell^{-1} day^{-1}	[3]
μ_C	Natural death rate of CTLs	2.0×10^{-2} day^{-1}	[3, 10, 16]

To affirm the sensitivity results we obtained with PRCC method, we further performed the model sensitivity using the eFAST method by implementing a MATLAB code developed by Kirschner and colleagues. For a given parameter i, we calculated the First-order (S_i) and total-order (S_{Ti}) sensitivity indices. The First-order sensitivity index indicates the fraction of model output variance that can be explained by the variance of a given parameter (input), while the total-order sensitivity index for a given input indicates the variance of the given model output that remains when all variances caused by other inputs, and covariances between all combinations of inputs, is removed. In eFAST, all model parameters (inputs) are varied within specific ranges at different frequencies, and the model solutions (outputs) are calculated. As in PRCC method, the model parameter values were allowed to vary from $1/2$ to twice its baseline values listed in Table S1 in the Supplementary Material, except for $\delta_{p/s}$ which is should be chosen from 0 and 1. To determine which parameter has a greatest influence in the model solutions, the Fourier transform of the model solutions is computed with respect to the amplitude of each parameter's frequency. Of note, the total-order sensitivity index is used as a measure of the global sensitivity, which accounts for the second and higher-order interactions between multiple model parameters. To test the statistical significance of each parameter, eFAST compares the significance of each parameter to a dummy variable (which is not included in the model equations, and does not affect model predictions in any way). Parameters that have a total-order sensitivity index that is less than or equal to that of the dummy

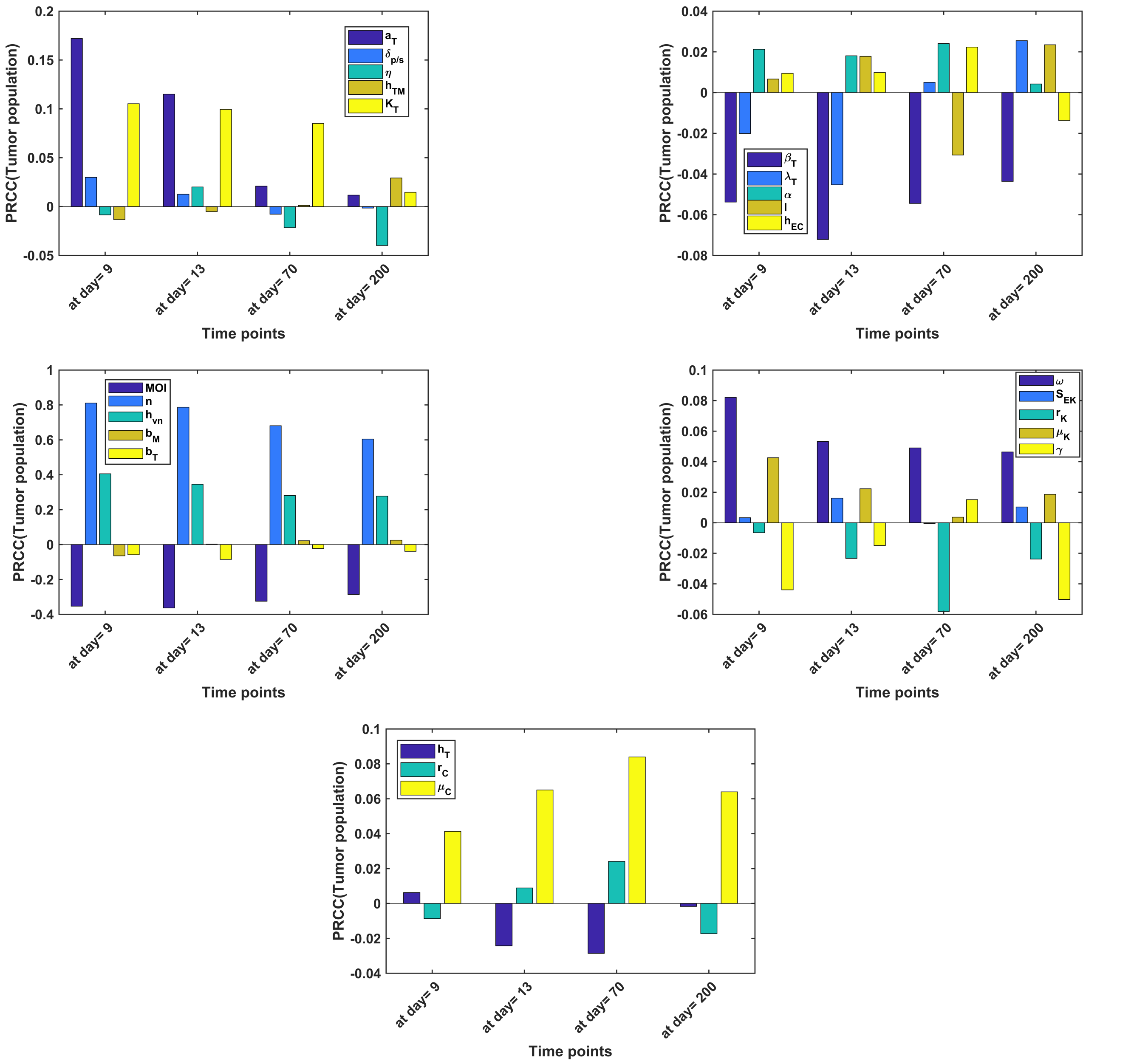

Figure 1. Tumor cell population sensitivity using PRCC. PRCC values for the model parameters using the tumor cell population as the variable (output) of interest. Statistically significant PRCC values (PRCC $> |0.5|$) for tumor cell population ($T_{tumor}(t) = T_u(t) + T_i(t)$) at days $9, 13, 70$ and 200

variable are regarded not significantly different from zero. The eFAST indices for our model parameters are shown in Fig. 2.

Fig. 2(A), the first-order index S_i, reveals that the multiplicity of infection (MOI) is the most significant parameter during the early stages (at days 9 and 13) of tumor growth, whereas the virus clearance, ω, and the rate of CTLs recruitment, γ, are most significant at latter time points. We also note that while other parameters have large eFAST total-order indices, S_{Ti}, at latter tumor growth stages, the MOI is the most consistently influential parameter at all time points (Fig. 2(B)).

Tumor growth in the absence of therapy and in the presence of strong immune response

Fig. 3 shows the dynamics of tumor growth in the absence of therapy and in the presence of strong immune response.

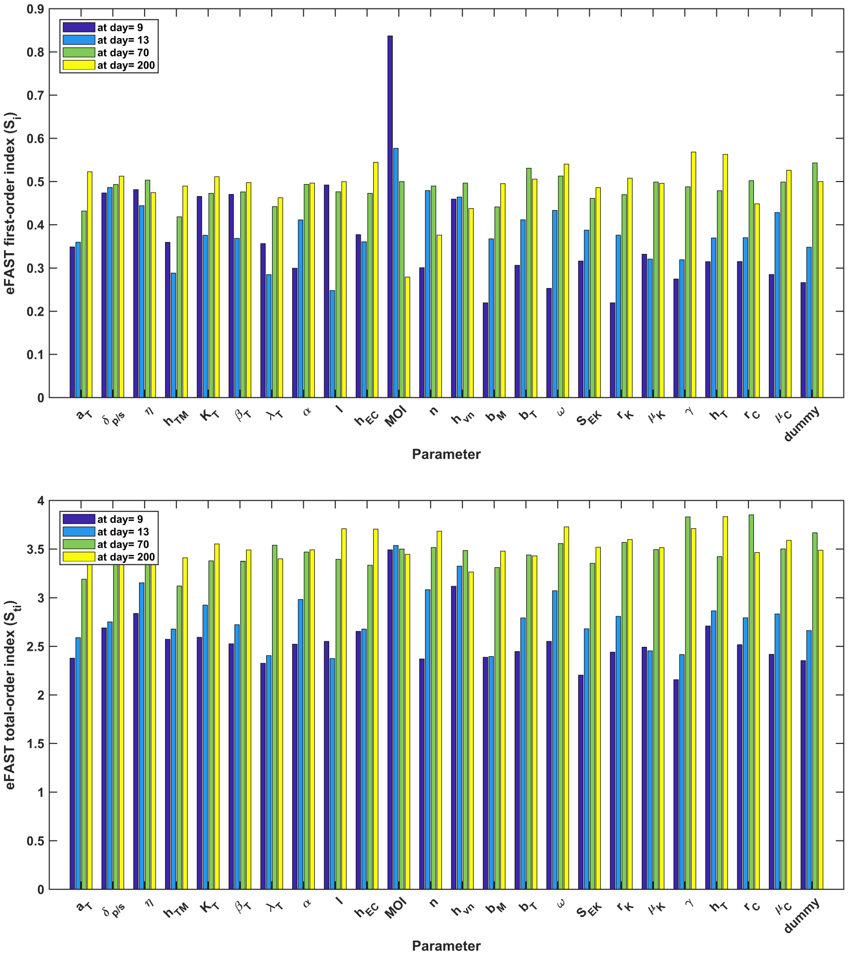

Figure 2. Tumor cell population sensitivity using eFAST. The first-order (S_i) index quantifies variance of the model prediction (output) with respect to variance of each individual model parameter (inputs) (A). The total-order sensitivity index quantifies the variance of the given model output that remains when all variances caused by other paramters, and covariances between all combinations of inputs, is removed (B). Here, the tumor cell population is taken as the model variable (output variable) of interest.

oAd-MSC promotive/suppressive-associated results

In this section, we further demonstrate how the oAd-MSC promotive/suppressive effects may impact treatment outcomes by arbitrarily choosing $\eta = 1,6$ and 8 for demonstrative purposes. Fig. 4 shows the influence of increasing the absolute value of η on model output.

Multiplicity of infection (MOI)-associated results

In this section, it is demonstrated that increasing the multiplicity of infection (MOI) on MSCs correlates with improved treatment outcomes. Although the precise MOI values are often determined experimentally, Fig. 5 shows the impact of increasing MOI on carrier cells (mesenchymal stem cells).

Comparison of oncolytic Ad-based therapies-associated results

From Fig. 5 in our main paper, we noted the following simulations results on day 35 of each therapy, summarized in Table S2.

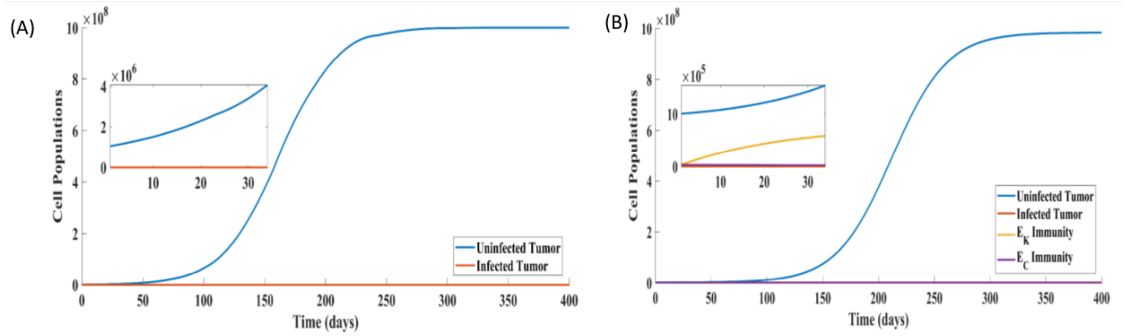

Figure 3. **Tumor growth in the absence of therapy and in the presence of strong immune response.** **(A)** Unperturbed tumor growth in a representative initial population of $T_u = 10^6$ cells. **(B)** shows how each tumor grows over time under immune response in the absence of oAd-MSC therapy, while **(B)** indicates tumor regression profile in the case where MSCs are not used as delivery vehicles and the immune response is present within tumor micorenvironment. In **(A)** and **(B)** the model is run for a long time-period of 400 days to reveal the longer-term behavior of the model dynamics, and the inset figure shows a detailed view of the model dynamics for a short time period of 35 days, as in the experiments. **(C)** Simulated growth of uninfected tumor cells in the presence of strong immune response within the tumor microenvironment. The initial conditions were taken as $T_{u0} = 1 \times 10^6$ cells, $E_{K0} = 9 \times 10^3$ cells, and $E_{C0} = 1 \times 10^3$ cells.

Table S2. **Comparison of oAd-MSC therapy and the direct dose of oncolytic Ad therapy.** Total tumor cell population at the end of therapy (day 35)

Probability ($\delta_{p/s}$)	Therapy	Total tumor cells (Tumor promotion)	Total tumor cells (Tumor suppression)
0.1	No therapy	1.389759×10^8	1.389759×10^8
	Naked oncolytic Ad	6.892915×10^6	6.892915×10^6
	oAd-MSC	6.947258×10^6	5.975098×10^6
0.5	No therapy	1.389759×10^8	1.389759×10^8
	Naked oncolytic Ad	6.892915×10^6	6.892915×10^6
	oAd-MSC	8.355634×10^6	3.888177×10^6
0.9	No therapy	1.389759×10^8	1.389759×10^8
	Naked oncolytic Ad	6.892915×10^6	6.892915×10^6
	oAd-MSC	9.076734×10^6	2.199592×10^6

Appendix A: Initial conditions and parameter estimates

The initial conditions of the model follow the experimental protocol for the *in vivo* setting described in the Supplementary section: **Experiments** and in Yoon et. al[27]. Since the virus replication of oncolytic Ads in MSCs was not assessed for the *in vivo* setting, we shall use the assessment made *in vitro* to estimate the virus production at day 5 post-incubation with MSCs. We denote by $V^\star$ the number of virions recovered at day 5 post-infection of the oAd-MSCs, at 10 MOI *in vitro*, and we assume that this represents the tentative number of virions to be released into the tumor microenvironment. Note that an amount of oAd-MSCs (1×10^6 MSCs infected with 5×10^8 VP of oncolytic Ad) is injected intravenously on day 9 and 13. This is modelled according to the function $u(t) = u_0(\delta(t-9) + \delta(t-13))$, where $u_0 = 1 \times 10^6$ cells and $\delta(t)$ is the Dirac delta function. On the other hand, when the oncolytic Ads are directly injected into the system, we use the value of $V_0 = 5 \times 10^8$ virus particles (VP) of oncolytic Ad as in the experiments described above. Model parameters that are taken from the available literature, in Table S1, were regarded as global free parameters for both model fitting and simulations, whereas the remaining unknown parameters were estimated from related treated tumor growth data and oAd-MSC data in Yoon et. al[27]. As explained earlier, the virus was administered on days $t = 9$ and $t = 13$, whereas the tumor was measured one week before. We set the initial tumor size before treatment, T_{u0}, to the corresponding tumor size at $t = 7$, which is the predicted size of the untreated tumor by the logistic equation, Eq. 1, in our model. Additionally, photon flux from the tumor is directly proportional to the number of live cells expressing luciferase; thus, the bioluminescence signal intensity correlates with tumor size (indicated by cell volume). We, therefore, assumed that the total tumor population $T_u(t) + T_i(t)$ (total tumor volume) is the sum of the volumes of each cell from regions of interest. We converted tumor volumes into tumor population (cells) by assuming that $1mm^3$ contains 1×10^6 tumor cells. The rest of the initial conditions are: $T_{i0} = 0$ cells, $E_{K0} = 0$ cells, and $E_{C0} = 0$ cells.

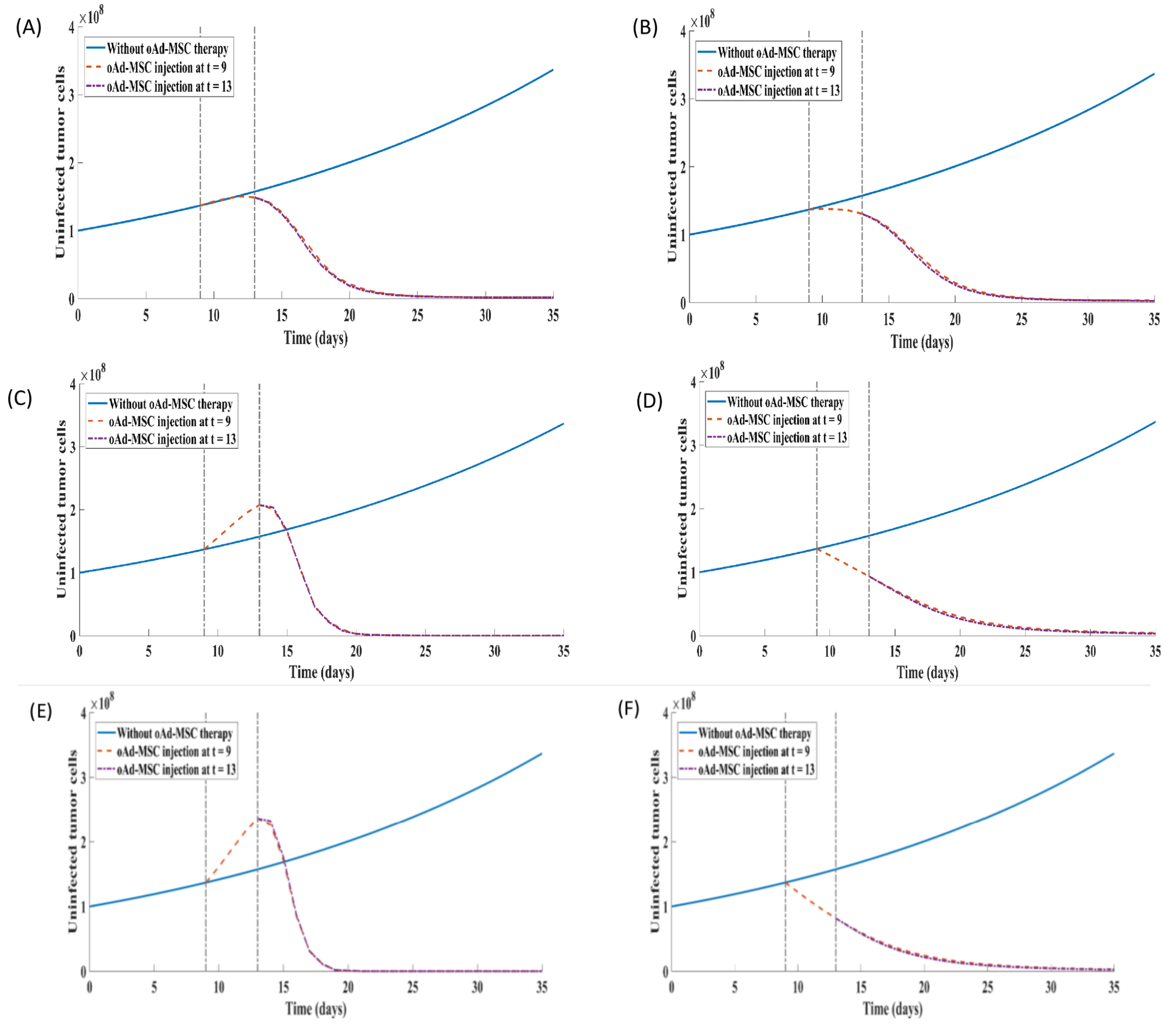

Figure 4. The simulated impact of tumor promotion or suppression by oAd-MSCs. A low dosage of 1×10^6 oAd-MSCs is injected into the system on day 9 and 13. These results are obtained by varying the values of η, while other parameters remained fixed in Table S1. **(A)** and **(B)** illustrate tumor growth for $\eta = 1$ in the case where oAd-MSCs promote or suppress tumor proliferation. **(C)** and **(D)** respectively indicate tumor growth for $\eta = 6$ in the case where oAd-MSCs promote or suppress tumor proliferation. The last two figures, **(E)** and **(F)**, respectively demonstrate tumor growth trajectories for $\eta = 8$ in the case where oAd-MSCs promote or suppress tumor proliferation.

Using our experimental datasets, we estimated the values of some unknown model parameters (for instance, see Figs. S7 and S8). We now describe the approaches we used to estimate the unknown parameters in our model. We determine the unknown parameter values in the model by either fitting the appropriate model equation(s) to the available experimental data or considering plausible biological ranges from several sources in the literature.

The uninfected tumor. We estimate that one oAd-MSC interacting with one tumor cell over one day prior to its lysis by oncolytic viruses has a 50% chance of either promoting or suppressing tumor cell growth. Hence, the corresponding probability coefficient, $\delta_{p/s}$, is 0.5. The tumor growth promotive/suppressive constant, $\eta = 0.45$, and the half-saturation constant that supports half-maximum tumor growth or suppression, $h_{TM} = 500$, are *ad hoc* values and were chosen to give possible biological outcomes.

The infected tumor. To estimate the net growth rate of tumor cells, we used control (PBS) data from Figure 5(B) from[27], a data set representing Hep3B cells treated with PBS on days 9 and 13. We fit Equation 1 of our model, logistic growth, that describe tumor growth in the absence of treatment. All model parameters were set to zero, except for the net tumor growth

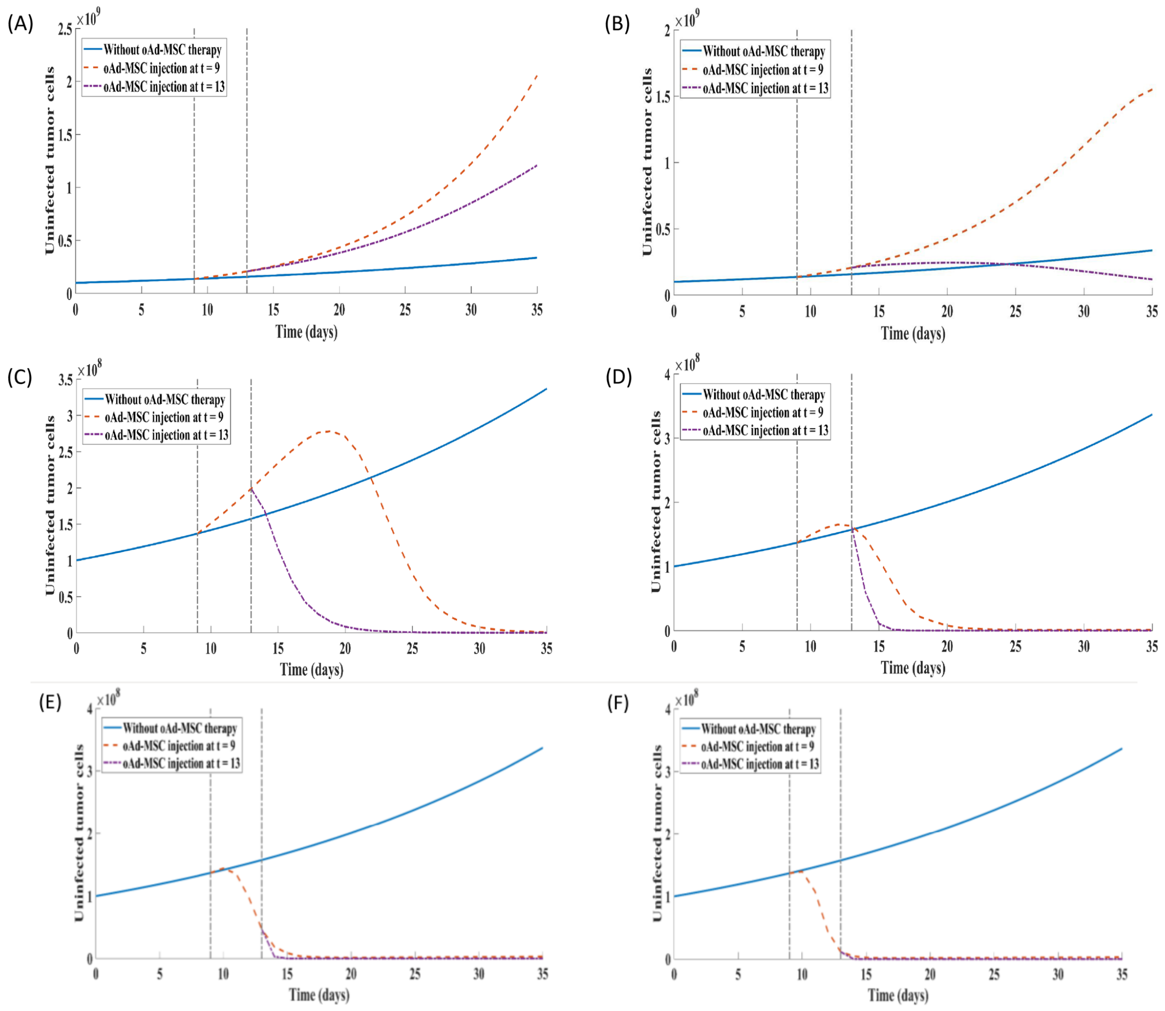

Figure 5. The effect of loading MSCs with different MOIs on tumor cell lysis. Here, a high dosage of 1×10^8 oAd-MSCs is injected into the system on day 9 and 13 for each MOI. All simulations were conducted under the case where oAd-MSCs are assumed to promote tumor cell proliferation. **(A)** indicates tumor growth at MOI 1. **(B)** shows the simulated tumor growth at MOI 2. **(C)** elicits tumor growth profile at MOI 5. Tumor growth trajecties at MOI 10 are shown in **(D)**. The last two figures, **(E)** and **(F)**, respectively illustrate tumor growth trajectories at MOI 20 and 50. The parameters chosen for these simulations were as followings: $a_T = 0.035$[26], $K_T = 5.14 \times 10^{11}$[26], and other parameters were unchanged from Table S1.

rate, a_T, which was to be estimated from the data. We estimated a_T using a non-linear least-squares fit of the log of the tumor data. We obtained the fit $a_T = 0.315$/day and retained this estimate for the remaining subsequent simulations. We also estimated the infection rate of tumor cells (β_T) by oncolytic Ad using Equation 1 and treated tumor growth data, and obtained $\beta_T = 12.8 \times 10^{-4}$ virion^{-1} day^{-1}. See Figure 3 in the main article.

The oAd-MSC lysis. By minimizing the sum of squares error (SSE) between the oncolytic Ad loaded MSC (oAd-MSC) data points and the lysis function estimates using the MATLAB function *lsqcurvefit*, we used a non-linear least-squares fitting procedure to fit the oAd-MSC data for day 2,5 and 7, respectively. We proposed the exponential response function of the form $b(1)exp(b(2)x) + b(3)$, where $b(1)$ and $b(2)$ are parameters related to n and h_{vn} of the Hill-function in equation 9, $b(3)$ is a constant, and x is the corresponding oAd-MSC data for days 2,5 and 7. Representative example fits to oAd-MSC data for all MOIs are shown in Fig 6. In this fitting, we considered a constant initial administration of oAd-MSC (1×10^6 MSCs infected with 5×10^8 VPs). However, comparison of the parameter estimates for oAd-MSC lysis shows that the Hill-coefficient, n, is an important variable and determinant of the outcome of MSC viability (see Fig 6). Given the small variability in the MOIs injected, it is unlikely that the MSC viability from the different mice had significant variability as a result of MSC permissiveness to virus replication or Ad oncolysis. Thus, we expect the MSC lysis parameters to be similar for all days. In

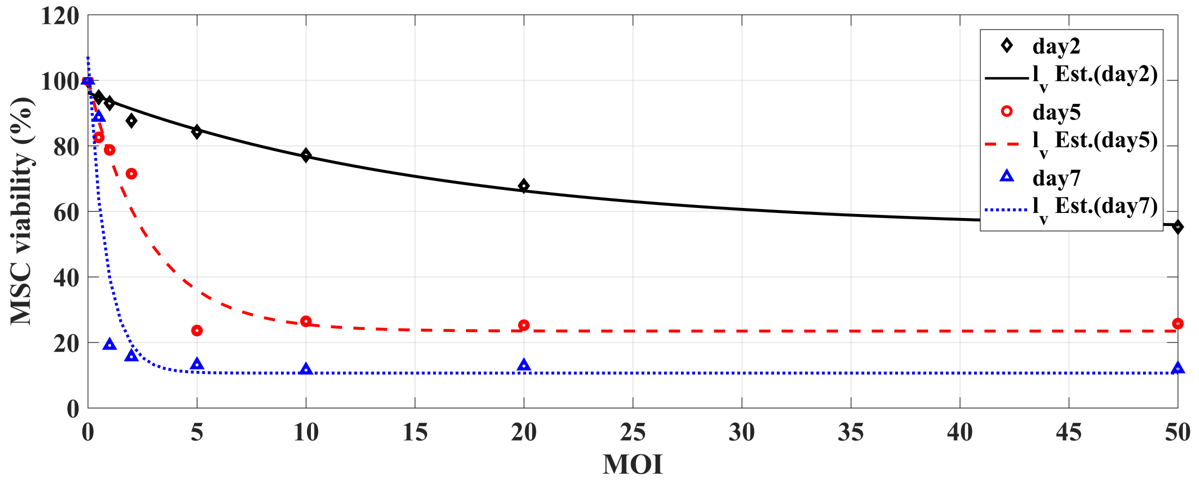

Figure 6. Fits to MSC cell viability data. oAd-MSC lysis is a MOI-dependent variable. For all plots, $h_{vn} = 20$ is constant, and the estimates for the Hill-coefficient, n, are $1, 0.6, 0.64$ for day $2, 5$ and 7, respectively.

Fig 6, we also notice that there exists good agreement between the experimental data and lysis function prediction with respect to the average decline in oAd-loaded MSCs, but as expected, different Hill-coefficients, n.

The oncolytic virus. The virus burst sizes, b_M, is determined by dividing the infectious progeny viruses produced from all infected cells by the initial number of cells[28,29]. For example, at MOI 10, $b_M = \frac{V^\star}{M_{i0}} = \frac{266385744}{1\times 10^6} = 266$ plaque forming units (pfu), where $V^\star$ is the average virus yields recovered from all infected cells at day 5 post-infection of the oAd-MSCs, and M_{i0} is the number of infected oAd-MSCs.

Appendix B: Tumor evolution in the absence of therapy but in the presence of the immune response

In the absence of therapy ($\xi_M = \xi_V = 0$) but in the presence of the immune response, the system Eqs (1 - 6) in the main article now reduces to three core equations:

$$\frac{dT_u}{dt} = a_T T_u\left(1 - \frac{T_u}{K_T}\right) - \lambda_T E_K T_u - d\left(\frac{(E_C/T_u)^l}{h_{E_C} + (E_C/T_u)^l}\right) T_u \tag{1}$$

$$\frac{dE_K}{dt} = S_{E_K}(t) - r_K \lambda_T E_K T_u - \mu_K E_K \tag{2}$$

$$\frac{dE_C}{dt} = \gamma E_C \frac{T_u}{h_T + T_u} - r_C E_C T_u - \mu_C E_C. \tag{3}$$

We note that the exponent l, which represents how the lysis rate depends on the CTL/tumor ratio, may produce results which may fail to preserve positivity of model solutions. This occurs whenever $l > 1$. Note also that in all our simulations we used $l = 2/3 < 1$ as in[4].

Appendix C: Tumor evolution in both the presence of OVs (without MSCs as delivery vehicle) and the immune response

In this case, oncolytic Ads are directly injected into the system (i.e., $(\xi_M = 0, \xi_V = 1)$). Thus, the system Eqs (1 - 6) in the main article reduces to five equations:

$$\frac{dT_u}{dt} = a_T T_u \left(1 - \frac{T_u}{K_T}\right) - \lambda_T E_K T_u - d\left(\frac{(E_C/T_u)^l}{h_{E_C} + (E_C/T_u)^l}\right) T_u \tag{4}$$

$$\frac{dT_i}{dt} = \beta_T(t_i) T_u V - l_v(t_i, MOI) T_i - \lambda_T E_K T_i - d\left(\frac{(E_C/T_u)^l}{h_{E_C} + (E_C/T_u)^l}\right) T_i \tag{5}$$

$$\frac{dV}{dt} = \xi_V u_V(t) + l_v(t_i, MOI) b_T T_i - \omega V \tag{6}$$

$$\frac{dE_K}{dt} = S_{E_K}(t) - r_K \lambda_T E_K T_u - \mu_K E_K \tag{7}$$

$$\frac{dE_C}{dt} = \gamma E_C \frac{T_u}{h_T + T_u} - r_C E_C T_u - \mu_C E_C. \tag{8}$$

References

1. Kim, Y. *et al.* Complex role of NK cells in regulation of oncolytic virus–bortezomib therapy. *PNAS* **115**, 4927–4932 (2018).
2. de Pillis, L. & Radunskaya, A. A mathematical model of immune response to tumor invasion. In *Computational Fluid and Solid Mechanics*, 1661–1668 (ed. K.J. Bathe (Elsevier Science Ltd), 2003).
3. de Pillis, L., Radunskaya, A. & Wiseman, C. A validated mathematical model of cell-mediated immune response to tumour growth. *Cancer Res.* **65**, 7950–7958 (2005).
4. de Pillis, L., Gallegos, A. & Radunskaya, A. A model of dendritic cell therapy for melanoma. *Mol. Cell. Oncol.* **3**, 1–14 (2013).
5. de Pillis, L., Caldwell, T., Sarapata, E. & Williams, H. Mathematical modeling of regulatory T cell effects on renal cell carcinoma treatment. *AIMS* **18**, 915–943 (2013).
6. Bhat, R. & Rommelaere, J. Emerging role of natural killer cells in oncolytic virotherapy. *Immunotargets Ther.* **4**, 65–77, DOI: 10.2147/ITT.S55549 (2015).
7. Yamano, T. *et al.* Whole cell vaccination using immunogenic cell death by an oncolytic adenovirus is effective against a colorectal cancer model. *Mol. Ther.* **3**, 16031, DOI: 10.1038/mto.2016.31 (2016).
8. Marcus, A. *et al.* Recognition of tumors by the innate immune system and natural killer cells. *Adv. immunology* **122**, 91–128, DOI: 10.1016/B978-0-12-800267-4.00003-1 (2014).
9. Farnault, L., Sanchez, C., Baier, C., Treut, T. L. & Costello, R. Hematological malignancies escape from NK cell innate immune surveillance: mechanisms and therapeutic implications. *Clin. Dev. Immunol.* **2012**, 1–8, DOI: 10.1155/2012/421702 (2012).
10. Mahasa, K., Ouifki, R., Eladdadi, A. & de Pillis, L. Mathematical model of tumor–immune surveillance. *J. Theor. Biol.* **404**, 312–330, DOI: 10.1016/j.jtbi.2016.06.012 (2016).
11. Eftimie, R., Dushoff, J., Bridle, B., Bramson, J. & Earn, D. Multi-stability and multi-instability phenomena in a mathematical model of tumor-immune-virus interactions. *Bull. mathematical biology* **73**, 2932–2961 (2011).
12. Okamoto, K., Amarasekare, P. & Petty, I. Modeling oncolytic virotherapy: Is complete tumor-tropism too much of a good thing? *J. theoretical biology* **358**, 166–178, DOI: 10.1016/j.jtbi.2014.04.030 (2014).
13. Mahasa, K., Eladdadi, A., de Pillis, L. & Ouifki, R. Oncolytic potency and reduced virus tumor-specificity in oncolytic virotherapy. A mathematical modelling approach. *PLoS ONE* **12**, e0184347 (2017).
14. de Pillis, L., Caldwell, T., Sarapata, E. & Williams, H. Mathematical modeling of the regulatory T cell effects on renal cell carcinoma treatment. *Discret. Continuous Dyn. Syst. Ser.* **18**, 915–943 (2013).
15. Kim, P., Crivelli, J., Choi, I., Yun, C. & Wares, J. Quantitative impact of immunomodulation versus oncolysis with cytokine-expressing virus therapeutics. *Math. biosciences engineering* **12**, 841–858 (2015).
16. de Pillis, L., Gu, W. & Radunskaya, A. Mixed immunotherapy and chemotherapy of tumours: modeling, applications and biological interpretations. *J. Theor. Biol.* **238**, 841–862 (2006).
17. de Pillis, L. *et al.* Mathematical model creation for cancer chemo-immunotherapy. *Comput. Math. Methods Medicine* **10**, 165–184 (2009).
18. Marino, S., Hogue, I., Ray, C. & Kirschner, D. A methodology for performing global uncertainty and sensitivity analysis in systems biology. *J. theoretical biology* **254**, 178–196 (2008).
19. Saltelli, A. *et al.* *Global Sensitivity Analysis: The Primer* (Wiley, New York, 2008).
20. Saltelli, A. & Marivoet, J. Non-parametric statistics in sensitivity analysis for model output: a comparison of selected techniques. *Reliab. Eng. & Syst. Saf.* **28**, 229–253 (1990).
21. Ratto, M., Pagano, A. & Young, P. State dependent parameter metamodelling and sensitivity analysis. *Comput. Phys. Commun.* **177**, 863–876 (2007).
22. Prestwich, R. *et al.* The case of oncolytic viruses versus the immune system: waiting on the judgment of solomon. *Hum. gene therapy* **20**, 1119–1132 (2009).
23. VanSeggelen, H., Tantalo, D., Afsahi, A., Hammill, J. & Bramson, J. Chimeric antigen receptor–engineered T cells as oncolytic virus carriers. *Mol. Ther. – Oncolytics* **2**, 150014, DOI: 10.1038/mto.2015.14 (2015).

24. Mader, E. *et al.* Mesenchymal stem cell carriers protect oncolytic measles viruses from antibody neutralization in an orthotopic ovarian cancer therapy model. *Clin. Cancer Res.* **15**, 7246–7255 (2009).

25. Ong, H. *et al.* Systemically delivered measles virus-infected mesenchymal stem cells can evade host immunity to inhibit liver cancer growth. *J. hepatology* **59**, 999–1006 (2013).

26. Walker, R. *et al.* Immune interconnectivity of anatomically distant tumors as a potential mediator of systemic responses to local therapy. *Sci. reports* **8**, 9474 (2018).

27. Yoon, A. *et al.* Mesenchymal stem cell-mediated delivery of an oncolytic adenovirus enhances antitumor efficacy in hepatocellular carcinoma. *Cancer research* canres–3900, DOI: 10.1158/0008-5472.CAN-18-3900 (2019).

28. Garcß́a-Castro, J. *et al.* Treatment of metastatic neuroblastoma with systemic oncolytic virotherapy delivered by autologous mesenchymal stem cells: an exploratory study. *Cancer gene therapy* **17**, 476–483 (2010).

29. Shashkova, E., May, S. & Barry, M. Characterization of human adenovirus serotypes 5, 6, 11, and 35 as anticancer agents. *Virology* **394**, 311–320 (2009).
